# Collagen Type VI Alpha 1 as a Regulator of Redox Homeostasis in Antioxidant‐Enhanced Osteogenesis of Dental Stem Cells

**DOI:** 10.1111/cpr.70220

**Published:** 2026-05-07

**Authors:** Zhaosong Meng, Jiacheng Liu, Yue Zhang, Ruimeng Yang, Jingyi Zhang, Haosun Yang, Zheng Wang, Ran Wei, Zhe Li, Shuling Guo, Lizhi Hu, Lei Sui

**Affiliations:** ^1^ Department of Oral and Maxillofacial Surgery, Tianjin Medical University School and Hospital of Stomatology, Tianjin Key Laboratory of Oral Soft and Hard Tissues Restoration and Regeneration Tianjin Medical University Institute of Stomatology Tianjin People's Republic of China; ^2^ Department of Prosthodontics, Tianjin Medical University School and Hospital of Stomatology, Tianjin Key Laboratory of Oral Soft and Hard Tissues Restoration and Regeneration Tianjin Medical University Institute of Stomatology Tianjin People's Republic of China; ^3^ Department of Geriatric Dentistry, Beijing Laboratory of Biomedical Materials Peking University School and Hospital of Stomatology Beijing People's Republic of China; ^4^ Key Laboratory of Immune Microenvironment and Disease (Ministry of Education), School of Basic Medical Science Tianjin Medical University Tianjin People's Republic of China

**Keywords:** alveolar bone regeneration, collagen type VI alpha 1, N‐acetylcysteine, PI3K‐AKT pathway, redox homeostasis, stem cell osteogenesis

## Abstract

Alveolar bone injury represents a prevalent clinical challenge in dentistry, for which stem cell‐based therapy has emerged as a promising strategy to promote bone regeneration. N‐acetylcysteine (NAC), a potent antioxidant, has been shown to modulate the PI3K‐AKT signalling pathway and potentially enhance osteogenesis; however, the specific downstream effectors mediating this process remain unidentified. In this study, post‐extraction serum metabolomic profiling revealed that alveolar bone injury is accompanied by systemic oxidative stress and metabolic remodelling. Transcriptomic analysis of antioxidant‐treated dental stem cells further identified type VI collagen A1 (COL6A1) as a key functional mediator. We subsequently investigated the role of COL6A1 in antioxidant‐mediated osteogenesis through immunofluorescence and protein assays, and performed knockdown and in vivo experiments to evaluate its function in oxidative stress regulation and osteogenic differentiation. Our results demonstrated that alveolar bone injury is associated with systemic oxidative stress and global metabolic alterations. In vitro, NAC markedly promoted the osteogenic differentiation of dental follicle stem cells (DFSCs) by activating the PI3K‐AKT pathway and upregulating COL6A1. COL6A1 knockdown resulted in elevated reactive oxygen species (ROS) levels, impaired mitochondrial function, and attenuated NAC‐mediated osteogenesis. In vivo, NAC‐treated DFSCs exhibited enhanced bone healing and extracellular matrix (ECM) deposition in a rat model of alveolar bone injury, effects that were mediated through COL6A1 upregulation. Collectively, these findings demonstrate that NAC enhances osteogenesis in DFSCs via the PI3K‐AKT‐COL6A1 axis, offering a promising antioxidant‐based strategy for stem cell therapies in bone regeneration. Moreover, COL6A1 is essential for maintaining redox homeostasis and represents a potential therapeutic target for improving regenerative outcomes.

## Graphical abstract

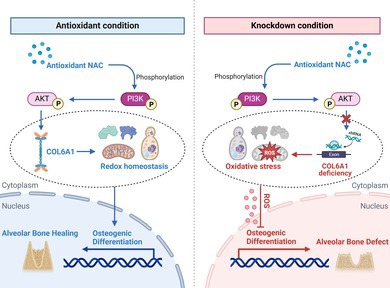

## 1 Introduction

Alveolar bone injury represents one of the most common and challenging clinical scenarios in dentistry, especially in oral and maxillofacial surgery. Current therapeutic strategies using autologous bone grafts and alternative biomaterials, with transplantation success largely contingent upon endogenous stem cells [1]. A systematic review of 27 preclinical studies indicates clinical benefits of stem cell‐based therapies for the regeneration of alveolar bone, especially in cases of functional stem cell depletion at injury sites [2]. Therefore, exogenous stem cell‐based therapy has become a potential choice to ensure well‐settled bone healing and the recovery of aesthetics and function.

Alveolar bone originates from the ectodermal germ layer through cranial neural crest cells, which developed into dental follicles—the developmental precursors of alveolar bone [3]. Consequently, dental follicle stem cells (DFSCs) are considered an optimal cell source for regenerating injured alveolar bone [4]. Bone healing facilitated by exogenous stem cells critically relies on the redox balance between reactive oxygen species (ROS) generation and the antioxidant system. An imbalance that favors an oxidative environment can induce cellular oxidative stress, impairing osteogenic capacity and bone healing processes [5, 6, 7]. Excessive ROS production is observed in various oral‐maxillofacial bone injuries, such as tooth extractions, cranial trauma, and implant osseointegration [8]. Studies showed that impairments in cells' intrinsic antioxidant defences contribute to oxidative stress induction [5]. Enhancing antioxidant capacity and preserving redox balance through ex vivo antioxidant treatment of stem cells is widely recognized for augmenting the clinical efficacy of stem cell therapies [5, 9]. While antioxidant‐conditioned stem cells have been employed in the regeneration of various tissues, their potential application in alveolar bone injury has been scarcely explored.

N‐acetylcysteine (NAC), a cysteine derivative, is extensively researched for its antioxidant properties. With high water solubility, NAC is quickly absorbed via anion exchange proteins on cell membranes [10]. NAC is postulated to serve as a disulfide bond reductant and ROS scavenger. It has undergone rigorous clinical investigation and has notably progressed to a phase IV clinical trial to evaluate its antioxidant efficacy [9]. Evidence suggests that NAC regulates excessive ROS levels to avert oxidative stress‐induced cellular damage and sustain appropriate redox homeostasis in stem cells. Notably, we and others found that NAC could relieve cellular oxidative stress and promote osteogenesis of mesenchymal stem cells in vitro and in vivo [11, 12, 13]. The PI3K‐AKT pathway serves as a pivotal regulator of cell growth and survival, with extensive research indicating its integral role in ROS regulation [14, 15]. Correspondingly, our group previously identified that both organic and inorganic antioxidants modulate ROS‐induced oxidative stress through the sequential activation of PI3K, a key component of the phospholipid kinase family [12, 16, 17]. However, the precise molecular targets of antioxidants warrant further investigation.

Since stem cell osteogenesis is commonly defined by a series of extracellular matrix (ECM) and osteogenic genes, the collagen family members are recognized as terminal effectors for cellular approaches to enhance bone healing [18, 19]. Although collagen proteins are tightly controlled by redox‐dependent processes, they are typically not involved in redox regulation. Collagen type VI alpha 1 (COL6A1) is one of the exceptions [20]. As a critical subunit of collagen VI, COL6A1 is a rate‐limiting factor in its production, present in the ECM of nearly all tissues. Distinct from other collagens, COL6A1 is redox‐sensitive and engages in redox regulation, attributed to cysteine residues in its triple‐helical domains [20]. It is reported that the pathological changes of COL6A1 deficiency are the result of higher ROS production [21]. Given the importance of COL6A1 in the maintenance of redox homeostasis and stem cell osteogenesis, COL6A1 may be a therapeutic target mediating the effect of antioxidants in stem cell‐based bone healing. Previous in vitro studies, using 3T3E1 preosteoblasts cell line, unravelled a function of COL6A1 in osteogenesis process [22], while studies with direct evidence in stem cells are still lacking so far.

This study highlights COL6A1 as a key downstream mediator in the PI3K pathway, improving the efficacy of stem cell therapy when applying antioxidant supplementation. Our findings indicate that COL6A1 is modulated by exogenous antioxidants and is essential for maintaining redox homeostasis and facilitating osteogenesis in dental stem cells, thereby highlighting its importance in promoting bone healing. More broadly, this study offers molecular insights into the mechanisms by which antioxidants confer pro‐healing properties in stem cell‐based therapies for alveolar bone regeneration.

## 2 Materials and Methods

### 2.1 Assessment of Plasma Redox Status

The inclusion criteria were (1) adults between 18 and 30 years of age (2) a pre‐existing indication for tooth extraction of impacted mandibular third molars (3) no history of smoking (4) no allergies, infections or autoimmune diseases (5) free of immunosuppressants or other drugs. All subjects were deemed healthy based on their medical history, physical examination, radiographic examination and routine blood testing. Peripheral blood samples were collected prior to the operation and 1 day after surgery from the Department of Oral and Maxillofacial Surgery of Tianjin Medical University Hospital of Stomatology. Samples were preserved in EDTA anticoagulation tubes and centrifuged at 3000 rpm for 10 min to isolate plasma. Malondialdehyde (MDA), hydrogen peroxide (H_2_O_2_), hydroxyl radical (·OH), superoxide anion radical (O_2_^·−^), total antioxidant capacity (T‐AOC), glutathione (GSH), superoxide dismutase (SOD) and catalase (CAT) were measured for determination of systemic redox status using the malondialdehyde assay kit, hydrogen peroxide assay kit, hydroxyl free radical assay kit, inhibition and produce superoxide anion assay kit, total antioxidant capacity assay kit, total glutathione assay kit, superoxide dismutase assay kit, catalase assay kit respectively as stated in the manufacturer's protocol (Nanjing Jiancheng Bioengineering Research Institute, Nanjing, China). This study was approved by the Ethics Committee of the Affiliated Hospital of Stomatology of Tianjin Medical University (permission no. TMUhMEC20230211) and performed in accordance with World Medical Association Declaration of Helsinki, ethical principles for medical research involving human subjects. Written informed consent was obtained from participants.

### 2.2 Plasma Metabolomics Analysis

Plasma samples were prepared following the above description. The extraction of metabolites and lipids was performed following standard procedures provided by Biomarker Technologies (Beijing, China). The LC/MS system for untargeted metabolomics and lipidomics is composed of Acquity I‐Class PLUS ultra‐high performance liquid tandem (Waters, MA, USA) and Xevo G2‐XS QTOF high resolution mass spectrometer (Waters, MA, USA). Metabolic analysis was performed in positive and negative ion modes at an electrospray ionization source. The raw data collected using MassLynx v4.2 (Waters, MA, USA) were processed by Progenesis QI software (Waters, MA, USA) for peak extraction, peak alignment and other data processing operations. Total metabolites and lipids were analysed in both positive and negative ion modes and annotated using Human Metabolome Database (HMDB; hmdb.ca/), Kyoto Encyclopedia of Genes and Genomes (KEGG; genome.jp/kegg/) and Lipidmaps (lipidmaps.org/). Differential analysis was performed using BMKCloud (biocloud.net/). Enrichment analysis based on Relational database of Metabolomics Pathways (RaMP; github.com/Mathelab/RaMP‐DB/), KEGG and Small Molecule Pathway Database (SMPDB; smpdb.ca/) was conducted by MetaboAnalyst 6.0 (metaboanalyst.ca/).

### 2.3 Isolation, Culture and Characterization of DFSCs

As previously described [12], rat DFSCs were isolated from unerupted mandibular first molars of Sprague–Dawley (SD) rat pups aged 7 days obtained from SPF Biotechnology Co. Ltd. (Beijing, China) and human DFSCs were isolated from fresh dental follicles from healthy individuals aged from 13 to 22 years old who underwent surgical extraction of unerupted third mandibular molars with surrounding low density of the dental follicle tissues observed on panoramic radiographs. Briefly, cells were digested by 0.1% collagenase type I and 1.5 U/mg dispase II, then cultured with minimum essential medium‐α (α‐MEM; Cytiva, MA, USA) containing 10% fetal bovine serum (FBS; HAKATA, Shanghai, China) and 1% penicillin/streptomycin (Solarbio, Beijing, China) in a humidified atmosphere of 5% CO_2_ at 37°C. Cells were collected with 0.25% trypsin with 1 mM EDTA‐4Na (Gibco, MA, USA) and washed with phosphate buffer solution (PBS; Solarbio, Beijing, China). Cells from passage 6 to 9 were used for the experiments. The expression of both surface and intracytoplasmic markers as well as the differentiation potential toward osteogenic, adipogenic and neural lineages were determined using passage 3 cells. Antibodies used for flow cytometry were as follows: FITC anti‐rat CD11b/c (1:100; BioLegend, CA, USA), FITC anti‐rat CD29 (1:100; BioLegend, CA, USA), PE anti‐rat CD45 (1:100; Elabscience, Wuhan, China), APC anti‐rat CD90 (1:100; Elabscience, Wuhan, China), PE anti‐rat CD106 (1:100; BioLegend, CA, USA), FITC anti‐human CD29 (1:100; Elabscience, Wuhan, China), APC anti‐human CD31 (1:100; Elabscience, Wuhan, China), FITC anti‐human CD44 (1:100; BioLegend, CA, USA), PE anti‐human CD90 (1:100; BioLegend, CA, USA), PE anti‐human CD117 (1:100; Elabscience, Wuhan, China), FITC mouse IgG1 κ isotype control (1:100; Elabscience, Wuhan, China), PE mouse IgG1 κ isotype control (1:100; Elabscience, Wuhan, China), APC mouse IgG1 κ isotype control (1:100; Elabscience, Wuhan, China), FITC mouse IgG2a κ isotype control (1:100; Elabscience, Wuhan, China), FITC armenian hamster IgG isotype control (1:100; Elabscience, Wuhan, China). This study was approved by the Animal Care and Use Committee of Tianjin Medical University and the Ethics Committee of the Affiliated Hospital of Stomatology of Tianjin Medical University (permission no. TMUSH‐hMEC2016082) and conducted according to the Declaration of Helsinki.

### 2.4 Treatment

NAC powder (Aladdin Chemical, Shanghai, China) was dissolved in 25 mM HEPES buffer (Solarbio, Beijing, China) and adjusted to pH 7 for the stock solution (500 mM). Cells were cultured in media with NAC at a final concentration of 5 mM. All antioxidants used in this study were listed in Table S1. The concentration of NAC was selected based on our preliminary screening studies, while the concentrations of the other antioxidants were determined according to previously published research [23, 24, 25, 26]. For PI3K inhibition, LY294002 (MCE, NJ, USA) dissolved in DMSO was added to media at a final concentration of 10 μM.

### 2.5 Rat and Experimental Surgery

This study was approved by the Animal Care and Use Committee of Tianjin Medical University and conformed to the ARRIVE guidelines 2.0. Following intraperitoneal anesthetization with 3% pentobarbital sodium (30 mg/kg), 6‐week‐old male SD rats received surgical extraction of the right maxillary first molar. The alveolar socket was formed using a 1# ball drill and filled with absorbable gelatin sponge (Xiang'en, Jiangxi, China) that was appropriate for the size of the socket as clinically indicated. Sponges were loaded with PBS vehicle, NAC solution, rat DFSC suspension (106 cells) or NAC‐treated cell suspension (106 cells) respectively depending on the randomized allocation of the animals. Based on this, rats were divided into four groups (*n* = 4 per group) after excluding a few animals with unsuccessful extraction. Tissue adhesive (*n*‐butyl cyanoacrylate; 3 M, MN, USA) was used to seal the socket wound. Rats in different groups were housed separately with the same conditions and no diet modifications were made for quality assurance. All animals survived until the end of the protocol and were humanely sacrificed by an overdose of anesthetics at postoperative weeks 1 and 3. Wound areas were manually contoured and analyzed by Image J (NIH, MD, USA).

### 2.6 Scanning Electron Microscopy (SEM)

The morphology of cells adhering to the gelatin sponge scaffold was examined by SEM. After fixation in 2.5% glutaraldehyde (Solarbio, Beijing, China) overnight at 4°C, cell‐seeded sponges were dehydrated in a graded ethanol solution series (from 20% to 100%). Samples were then critical‐point dried using liquid carbon dioxide and sputtered with a thin gold layer before examination under a Field Emission SEM Zeiss Gemini 500 (ZEISS, Jena, Germany).

### 2.7 Histological Analysis

The maxillary segments were dissected and fixed with 4% paraformaldehyde for 2 days. After decalcification with EDTA solution (Servicebio, Hubei, China) for 21 days, the specimens were embedded in paraffin (Leica, Wetzlar, Germany) and cut into 4‐μm‐thick slices. The sections were stained with haematoxylin‐eosin (HE; Servicebio, Hubei, China) for morphological examination and masson trichrome (Servicebio, Hubei, China) for ECM staining. PI3K‐AKT signalling pathway and osteogenic markers were detected using standard immunohistochemistry and immunofluorescence methods (Servicebio, Hubei, China). Images were captured using an optical microscope (Nikon, Tokyo, Japan) and a fluorescence microscope (Nikon, Tokyo, Japan). Antibodies were as follows: anti‐COL1A1 (1:500; ProteinTech, Wuhan, China), anti‐COL6A1 (1:500 for immunohistochemistry, 1:100 for immunofluorescence; ProteinTech, Wuhan, China), anti‐PI3K (1:200; Abclonal, Wuhan, China), anti‐AKT (1:200; ProteinTech, Wuhan, China), anti‐phospho‐PI3K (1:100; Zenbio, Chengdu, China), anti‐phospho‐AKT (1:200; ProteinTech, Wuhan, China), HRP‐conjugated anti‐rabbit IgG (1:200; Servicebio, Wuhan, China), HRP‐conjugated anti‐mouse IgG (1:200; Servicebio, Wuhan, China), Cy3‐conjugated anti‐rabbit IgG (1:300; Servicebio, Wuhan, China).

### 2.8 RNA‐Sequencing Analysis

RNA was extracted with TRIzol reagent (TransGen, Beijing, China) and a total RNA kit (TransGen, Beijing, China) following the manufacturer's instructions. RNA‐sequencing was conducted following the standard operational procedure (genomics.cn/) in collaboration with BGI (Shenzhen, China). The expression of genes was reflected by transforming mapped transcript reads to transcripts per million (TPM). Differential gene expression analysis was performed utilizing the Dr.Tom platform (BGI, Shenzhen, China). Significance levels were Bonferroni‐corrected. Gene set enrichment analysis (GSEA) was performed by WebGestalt (webgestalt.org/). Enrichment analysis of Gene Ontology (GO; geneontology.org/) and KEGG was carried out by Dr. Tom and KOBAS‐i (bioinfo.org/kobas/). Generic PPI and KEGG network analysis were performed using NetworkAnalyst (networkanalyst.ca/).

### 2.9 Immunofluorescence Cytochemistry

Cells grown on autoclaved coverslips were fixed with 4% paraformaldehyde (Solarbio, Beijing, China) for 15 min, permeabilized with 0.2% Triton X‐100 solution (Solarbio, Beijing, China) for 10 min and blocked in 1% bull serum albumin (BSA; Solarbio, Beijing, China) solution for 1 h. For immunofluorescent staining, primary antibodies and fluorescent‐conjugated secondary antibodies and DAPI (Vector, Burlingame, CA) were successively applied to the sections. Images were acquired with a fluorescence microscope (Zeiss, Oberkochen, Germany), and mean fluorescence intensity (MFI) was analysed using Image J JACoP plugin. Antibodies were as follows: anti‐Vimentin (1:100; ProteinTech, Wuhan, China), anti‐CK14 (1:200; ProteinTech, Wuhan, China), CoraLite594‐conjugated anti‐β‐III‐tubulin (1:200; ProteinTech, Wuhan, China), anti‐COL1A1 (1:100; ProteinTech, Wuhan, China), anti‐COL6A1 (1:50; ProteinTech, Wuhan, China), anti‐LC3 (1:250; ProteinTech, Wuhan, China), anti‐SQSTM1 (1:250; Abcam, MA, USA), Alexa Fluor 488‐conjugated anti‐rabbit IgG (1:2000; Immunoway, TX, USA), Alexa Fluor 594‐conjugated anti‐rabbit IgG (1:2000; Immunoway, TX, USA).

### 2.10 Western Blot

Cells were lysed in RIPA buffer (Solarbio, Beijing, China) with PMSF (Solarbio, Beijing, China) on ice. Protein concentration was determined using a BCA protein assay kit (Thermo Scientific, MA, USA). After heat denaturation in loading buffer (EpiZyme, Shanghai, China) at 100°C for 10 min, protein samples were electrophoresed with SDS‐PAGE and transferred to PVDF membranes (Immobilon, MA, USA). The membranes were blocked in 5% non‐fat milk in TBST buffer for 2 h, incubated with primary antibodies overnight at 4°C and secondary antibodies for 1 h at room temperature. Protein bands were visualized by ECL reagents (TransGen, Beijing, China) using a Tanon‐5200 system (Tanon, Shanghai, China). Band intensity was normalized to the relative GAPDH levels using Image J and provided in the figures. Antibodies were as follows: anti‐COL1A1 (1:1000; ProteinTech, Wuhan, China), anti‐COL6A1 (1:1000; ProteinTech, Wuhan, China), anti‐PI3K (1:200; Santa Cruz, CA, USA), anti‐AKT (1:2000; CST, MA, USA), anti‐phospho‐PI3K (1:1500; CST, MA, USA), anti‐phospho‐AKT (1:1500; CST, MA, USA), anti‐LC3 (1:1250; CST, MA, USA), anti‐SQSTM1 (1:20000; Abcam, MA, USA), anti‐COL3A1 (1:200; Santa Cruz, CA, USA), anti‐FN1 (1:200; Santa Cruz, CA, USA), anti‐FBN1 (1:500; Bioss, Beijing, China), anti‐RUNX2 (1:100; Santa Cruz, CA, USA), anti‐OPN (1:1000; ProteinTech, Wuhan, China), anti‐GAPDH (1:6000; Immunoway, TX, USA), anti‐mouse IgG (1:6000; Immunoway, TX, USA), anti‐rabbit IgG (1:6000; Immunoway, TX, USA).

### 2.11 Enzyme‐Linked Immunosorbent Assay (ELISA)

Plasma samples were prepared following the above description. The amounts of COL6A1 in the human plasma were detected with ELISA kits (FANKEW, Shanghai, China) according to the manufacturers' instructions. Briefly, both samples and standards were added to an enzyme‐linked immunosorbent assay (ELISA) plate. The plate was sealed with a plate film and incubated at 37°C for 30 min. Following the incubation, the plate was washed five times with a wash buffer. Subsequently, an enzyme‐labelled reagent was added to each well, and the plate was incubated again at 37°C for 30 min. After a second round of five washes, colour development solutions A and B were added to the wells. The plate was then incubated at 37°C for 10 min to allow colour development. Finally, a stop solution was added, and the optical density (OD) of each well was measured at 450 nm within 15 min of stopping the reaction.

### 2.12 Molecular Docking Analysis

After obtaining the stereo structures of NAC and PI3K isoforms (PIK3R1, PIK3R2, PIK3R3, PIK3CD) by Pubchem and Uniprot, respectively, the structures were processed using ADT Tools. Docking and scoring were performed by Autodock Vina 1.2.3. After the docking was completed, the results were visualized and analysed using PyMol 2.2.0 and Discovery Studio 2021, respectively. For docking with protein–protein computational models, the pre‐processing of proteins was performed using AutoDockTools 1.5.7 and Docking Web Server (GRAMM) was used for protein–protein docking. Finally, intermolecular binding patterns for protein–protein interactions were predicted and analysed by PyMol.

### 2.13 Analysis of Gene Expression Correlations

Gene expression data for normal human tissues were obtained from GTEx Portal (gtexportal.org/). GEPIA (gepia.cancer‐pku.cn/) was applied for gene expression correlation analysis based on Pearson's coefficients: *R* < 0.1 reflected negligible correlation, 0.1 ≦ *R* < 0.3 reflected weak correlation, 0.3 ≦ *R* < 0.5 reflected moderate correlation, *R* ≧ 0.5 reflected strong correlation.

### 2.14 Real‐Time Quantitative Polymerase Chain Reaction (RT‐qPCR)

After cells were harvested, total RNA was extracted. cDNA was synthesized with TransScript all‐in‐one first‐strand cDNA synthesis supermix (TransGen, Beijing, China). RT‐qPCR was performed using PerfectStart green qPCR supermix (TransGen, Beijing, China) and the 7500 Fast Real‐Time PCR system (Thermo Fisher, MA, USA). The housekeeping gene GAPDH was used to normalize the results using the ΔΔCt method. The primer sequences (Sangon, Shanghai, China) were listed in Table S2.

### 2.15 Lentivirus Transfection

Human COL6A1 shRNA lentiviral particles and control lentiviral particles were obtained from Santa Cruz Biotechnology (TX, USA) and the transfection was performed following the manufacturer's instructions. Briefly, DFSCs were added with lentiviral particles and 5 μg/mL polybrene (Santa Cruz, TX, USA) when approximately 50% confluent, and cultured overnight before replacing plain media. Puromycin (MCE, NJ, USA) at a final concentration of 4 μg/mL was added to culture medium and fresh puromycin‐containing medium was changed every 3 days until resistant colonies were identified. The COL6A1‐knockdown efficiency was validated by western blot and RT‐qPCR.

### 2.16 Cell Viability Assay

Cells were seeded at a density of 2500 cells per well in a 96‐well plate. After cultivation at days 1, 2, and 3, cells were rinsed with PBS to remove residual NAC and cell viability was quantified using a cell counting kit‐8 (CCK‐8; Solarbio, Beijing, China) following the manufacturer's protocol. After 90 min of incubation at 37°C, the microplate reader was used to measure absorbance at 450 nm.

### 2.17 Cell Morphology Staining

Cells grown on climbing slices overnight were fixed with 4% paraformaldehyde, permeabilized with 0.2% Triton X‐100 solution, and blocked in 1% BSA solution. Cytoskeleton F‐actin and nuclei were stained in the dark with phalloidin (Solarbio, Beijing, China) and DAPI, respectively. Images were captured with the fluorescence microscope. Cell areas indicated by F‐actin were performed using Image J.

### 2.18 Cell Proliferation Assay

Cell proliferation was assessed by immunofluorescence staining for Ki67. Cells in active proliferative phases were stained using anti‐Ki67 antibody (1:1000; Abcam, MA, USA) and DAPI. Images were captured with the fluorescence microscope. Ki67 relative density was quantified based on the ratio of Ki67‐positive cells to total cells.

### 2.19 Redox Assays

After 4‐day culture, cells were harvested for redox state detection. Intracellular ROS levels were detected using DCFH‐DA probe (Beyotime, Shanghai, China). MFI of each sample was detected by flow cytometry (BD, NY, USA) and analysed using FlowJo (BD, NY, USA). Fluorescence images were visualized with the fluorescence microscope. The activities of SOD and CAT were detected using a total superoxide dismutase assay kit (Beyotime, Shanghai, China) and a catalase assay kit (Beyotime, Shanghai, China), respectively following the manufacturer's recommendation.

### 2.20 Assessment of Mitochondrial and Autophagic Functions

Mitochondrial oxidative status was evaluated using MitoSOX Red (Yeasen, Shanghai, China) for in situ staining of O_2_^·−^ and MitoTracker Green (Beyotime, Shanghai, China) for mitochondrial labeling. Images were captured with the fluorescence microscope and MFI was quantified by Image J. The level of mitochondrial membrane potential, apoptosis, and mitochondrial calcium content were analysed with JC‐1 probe (Beyotime, Shanghai, China), Annexin V‐FITC/PI cell apoptosis detection kit (TransGen, Beijing, China), and Rhod^−2^ AM probe (Yeasen, Shanghai, China) respectively using flow cytometry and FlowJo. Autophagy was detected using an autophagy assay kit with MDC (Beyotime, Shanghai, China). MFI was measured by flow cytometry and FlowJo. Autophagic markers LC3 and SQSTM1 were co‐stained with mitochondria labelled with MitoTracker Red (Beyotime, Shanghai, China), respectively. Co‐localization coefficients were calculated in Image J using plugin JACoP.

### 2.21 Cell Stemness Assay

For the clonogenic assay, cells were seeded at a density of 100 cells per well in 12‐well plates and cultured for 2 weeks. Cells were fixed in 4% paraformaldehyde, and colony formation was visualized by 0.1% crystal violet (Solarbio, Beijing, China). Images were acquired using the inverted microscope. To determine the expression of stemness markers CD44 and CD90, cells were collected at 80% confluence, incubated with FITC anti‐human CD44 and PE anti‐human CD90 for 30 min in the dark, and analysed by flow cytometry and FlowJo.

### 2.22 ECM Deposition Assay

Picro‐sirius red staining was used to examine ECM deposition because of its highly specific binding to collagen. Cells were seeded at a density of 5 × 10^5^ cells per well in a 6‐well plate and cultured for 12 days. Then, cells were fixed and stained with 0.1% picro‐sirius red solution (Sigma‐Aldrich, MO, USA) for 1 h. Images were acquired using the inverted microscope. The staining was solubilized in 0.1 M sodium hydroxide (Sigma‐Aldrich, MO, USA), and the microplate reader was used to measure absorbance at 540 nm for semi‐quantification.

### 2.23 Induction of Osteogenic and Adipogenic Differentiation

When cells reached nearly 100% confluence, growth media were switched to different differentiation media to induce osteogenic and adipogenic differentiation respectively. The osteogenic induction medium was α‐MEM supplemented with 10% FBS, 1% penicillin/streptomycin, 10 mM Na‐β‐glycerophosphate (Solarbio, Beijing, China), 100 nM dexamethasone (Sigma‐Aldrich, MO, USA) and 50 μg/mL ascorbic acid (Solarbio, Beijing, China). At 3‐day osteogenic induction, alkaline phosphatase (ALP) staining was carried out using a BCIP/NBT colour development kit (Beyotime, Shanghai, China) and ALP activity was measured by an alkaline phosphatase assay kit (Beyotime, Shanghai, China). Protein concentration of each sample was determined at A280 using a Nanodrop2000 (Thermo Scientific, MA, USA). The absorbance was measured using a microplate reader (Tecan, Männedorf, Switzerland) at 405 nm. At 3‐week osteogenic induction, mineralized nodules were stained using 0.2% alizarin red S (ARS; Solarbio, Beijing, China). Then the precipitation was sufficiently dissolved by 10% cetylpyridine chloride (Solarbio, Beijing, China) and the absorbance was measured using the microplate reader at 542 nm. The adipogenic induction medium was α‐MEM supplemented with 10% FBS, 1% penicillin/streptomycin, 111 μg/mL IBMX (Solarbio, Beijing, China), 72 μg/mL indomethacin (Solarbio, Beijing, China), 5 μg/mL insulin (Aladdin Chemical, Shanghai, China) and 0.4 μg/mL dexamethasone. At 2‐week adipogenic induction, oil red O staining was carried out using a modified oil red O staining kit (Beyotime, Shanghai, China). Images were acquired using an inverted microscope (Zeiss, Oberkochen, Germany).

### 2.24 Micro‐Computed Tomography (Micro‐CT)

The specimens were scanned using a micro‐CT scanning system (SkyScan1276; BRUKER, MA, USA) at 10‐μm scaled image pixel size with the source voltage of 55 kV, the source current of 144 μA and the exposure of 1316 ms. Representative two‐dimensional section images were acquired by DataViewer (BRUKER, MA, USA) and three‐dimensional models were reconstructed by CTvox (BRUKER, MA, USA). Assessments were performed by masked examiners (Z.W and H.S.Y). CTan (BRUKER, MA, USA) was used to manually contour the extraction socket of the first molar and analyse bone volume/tissue volume (BT/TV), bone mineral density (BMD) and trabecular parameters (trabecular thickness, Tb.Th; trabecular number, Tb.N; trabecular separation, Tb.Sp) at a beginning grayscale index of 4.1.

### 2.25 Statistical Analysis

Numerical data were presented as mean ± standard error of mean (SEM). The determination of sample size was guided by power analysis and conformed to the principles of the 3Rs. Two‐tailed student *t*‐test was performed for pairwise comparison by GraphPad Prism 10 (GraphPad Software, CA, USA). Differences between groups were considered statistically significant at *p* < 0.05 and resulting *P* were indicated in all figures.

### 2.26 Role of Funders

The funders played no role in study design, data collection, data analyses, interpretation or writing of report.

## 3 Results

### 3.1 Alveolar Bone Injury Accompanies Systemic Oxidative Stress and Global Metabolic Changes

**FIGURE 1:**
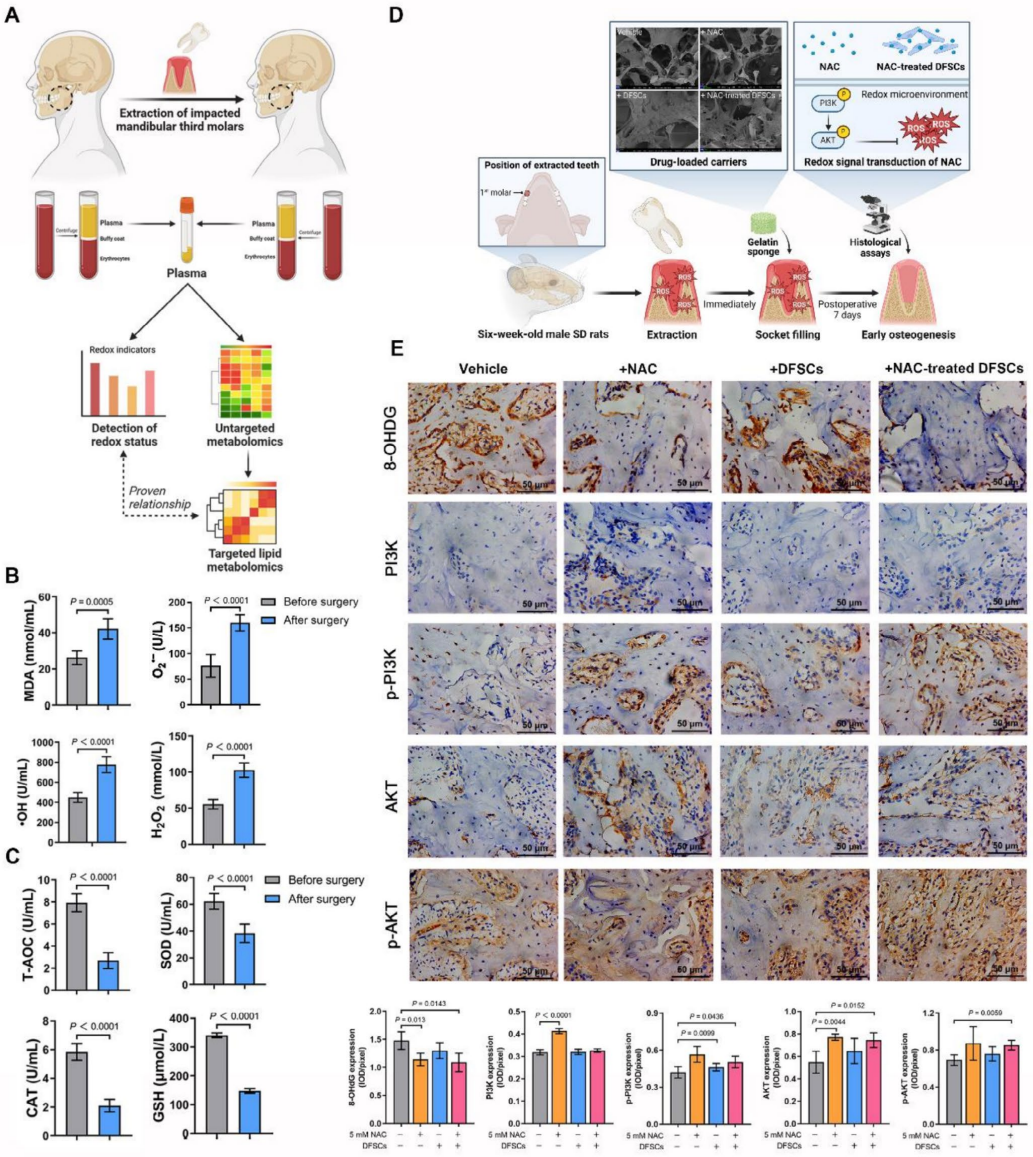
Alveolar bone injury accompanies systemic oxidative stress and global metabolic changes. (A) Schematic diagram of human plasma analysis after extraction of impacted mandibular third molar. (B) Plasma levels of oxidative indicators (MDA, O_2_^·−^, ·OH and H_2_O_2_) after extraction of impacted mandibular third molar. *p < 0.05 vs. control; *n* = 8 for each group. (C) Plasma levels of antioxidant (T‐AOC, SOD, CAT and GSH) after extraction of impacted mandibular third molar. *p < 0.05 vs. control; *n* = 8 for each group. (D) Representative diagram of NAC into DFSCs loaded gelatin sponge by scanning electron microscopy (SEM). Schematic diagram of NAC activates the PI3K‐AKT pathway to mitigate cellular oxidative stress and enhance early osteogenesis in alveolar sockets. (E) Representative immunohistochemistry staining and statistical analysis of key signalling molecules (PI3K, phosphorylated PI3K, AKT, phosphorylated AKT) and oxidative stress marker (8‐OHdG) in rats after treatment for 1 week. Scale bar, 50 μm.

Previous studies revealed that systemic oxidative stress resulting from alveolar bone injury occurred as early as 1 day after tooth extraction [27, 28]. Here, we designed the experimental workflow (Figure 1A) and initially validated the changes in redox status using plasma samples collected 1 day after surgery, compared to those prior to surgery. In the postoperative plasma samples, the levels of oxidative indicators (MDA, H_2_O_2_, OH and O_2_^·−^) were significantly elevated with a decrease in the antioxidant levels (T‐AOC, GSH, CAT and SOD) (Figure 1B,C). This redox perturbation suggested systemic oxidative stress caused by injuries.

Oxidative stress arises from an imbalance between ROS production and clearance pathways. Numerous studies have demonstrated the impact of intracellular ROS on the PI3K‐AKT signalling pathway [29], while others have shown the pathway's ability to regulate intracellular ROS levels [30]. Moreover, our previous studies also confirmed that exogenous antioxidant NAC could activate the PI3K‐AKT pathway to inhibit cellular oxidative stress and accelerate early osteogenesis in alveolar sockets in combination with stem cell‐based therapy (Figure 1D). Correspondingly, we further investigated the redox state and PI3K signals in alveolar sockets after antioxidant treatment in vivo. In a rat model of alveolar bone injury, gelatin sponges loaded with PBS, NAC, DFSCs, or NAC‐treated DFSCs were implanted into the alveolar sockets to measure the levels of ROS and the PI3K‐AKT signalling pathway in the nascent alveolar bone (Figure 1D). Immunohistochemical results showed that the expression of 8‐OHdG, an indicator of oxidative stress, significantly decreased after treatment with NAC or NAC‐treated DFSCs while the activation of the PI3K‐AKT pathway was observed in the NAC‐treated DFSCs group as indicated by the upregulation of phosphorylated PI3K (p‐PI3K), AKT and phosphorylated AKT (p‐AKT) (Figure 1E). From the above results, it was suggested that the PI3K‐AKT pathway participates in NAC‐mediated antioxidative regulation in the reparative regeneration of alveolar bone injuries, which may be closely related to stem cell osteogenesis. However, the molecular mechanisms beyond the antioxidant and pro‐osteogenic activity of NAC have not been thoroughly investigated so far.

To explore alterations accompanying redox imbalance, we performed the untargeted metabolomics for comprehensive metabolic profiles. Overall, a total of 2158 metabolites were annotated. The matching results were integrated with the HMDB and KEGG databases, showing that most of the metabolites belonged to lipids and lipid‐like molecules (Figure S1A) and were related to lipid metabolism (Figure S1B). Then, 194 differentially expressed metabolites (DEM) were defined as VIP > 1 and *P* < 0.05 (Figure S1C,D). DEMs with least *P* were associated with oxidative stress, such as oleoyl ethanolamide, choline, ascorbyl stearate and lysoPC (Figure S1E,F), while correlation existed among these metabolites (Figure S1G). RaMP enrichment results showed enrichment in multiple lipid metabolic pathways (Figure S1H) which was consistent with metabolite annotation. Analysis based on KEGG and SMPDB further revealed enrichment of glycerophospholipid metabolism and phospholipid biosynthesis respectively (Figure S1I,J). Oxidative stress is thought to be strongly linked with lipid metabolism [31]. We therefore used a targeted metabolomics method to study the changes in lipid metabolites. A total of 3442 lipids were annotated and most of them belonged to glycerolipids, glycerophospholipids and fatty acyls according to the Lipidmaps database (Figure S2A). 214 lipids were identified as DEMs (Figure S2B,C) and a significant proportion of them was glycerophospholipids (Figure S2D). Lipids with least *P* were also mostly glycerophospholipids (Figure S2E,F) and multiple significant correlations were observed among them (Figure S2G). In line with the aforementioned findings, RaMP enrichment of differential lipids showed that phospholipid biosynthesis was remarkably enriched with the smallest *P* (Figure S2H). Such phospholipid‐related metabolic signatures were likely associated with the accompanying oxidative stress in alveolar bone injury. PI3K, as a crucial signalling molecule in phospholipid metabolism, reinforces the previous in vivo findings by demonstrating its involvement in the regulation of oxidative stress, as supported by the aforementioned omics analysis results.

### 3.2 Treatment With NAC Possesses Pro‐Osteogenic Roles via COL1A1 and COL6A1

**FIGURE 2:**
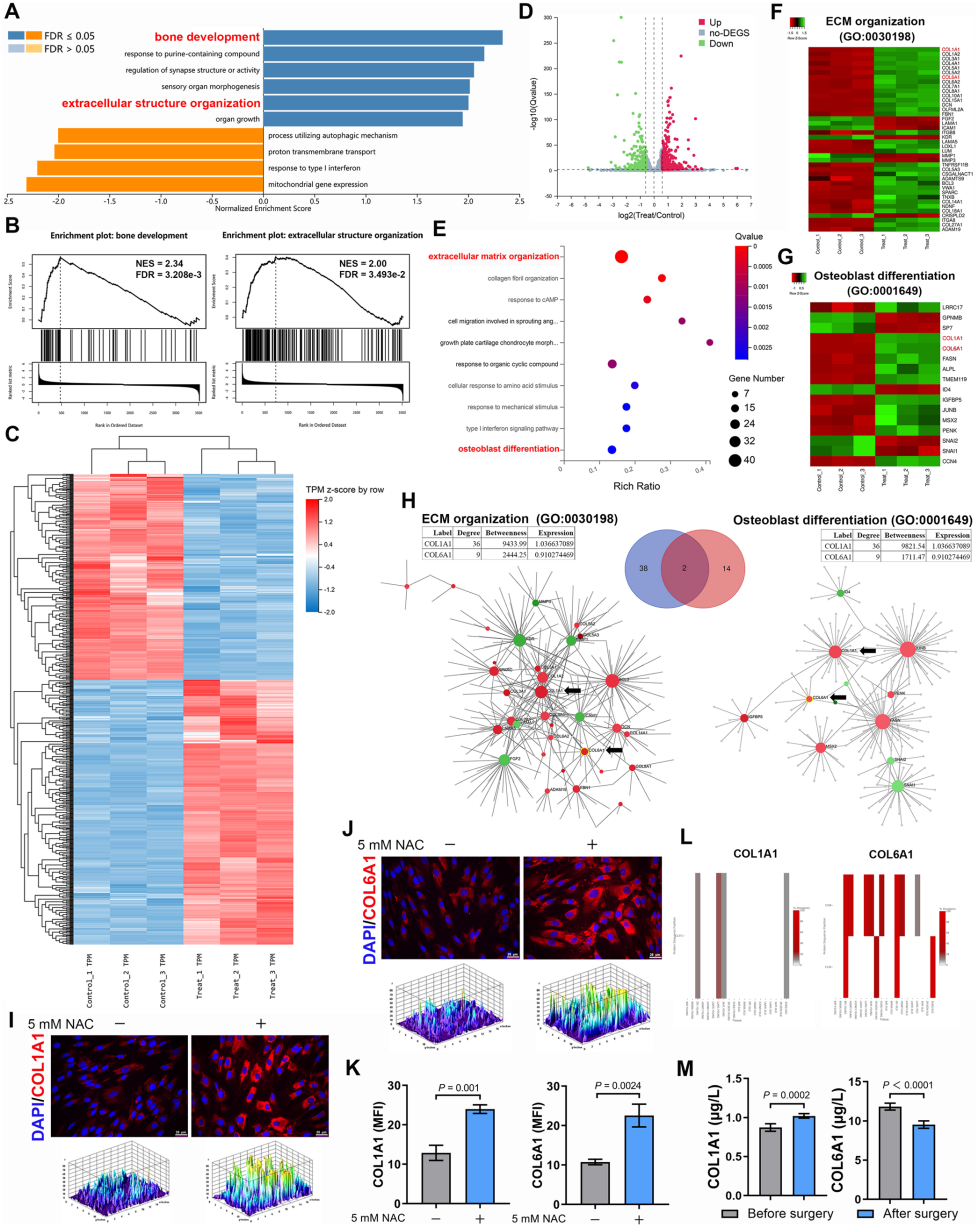
Treatment with NAC possesses pro‐osteogenic roles via COL1A1 and COL6A1. (A and B) Gene set enrichment analysis (GSEA) showing increased enrichment of ECM‐related and osteogenesis‐related genes in DFSCs treated by 5 mM NAC. (C) Heatmap of differentially expressed genes (DEGs) in DFSCs treated by 5 mM NAC. (D) Volcano plot of DEGs in DFSCs treated by 5 mM NAC. (E) Gene ontology (GO) enrichment analysis of DEGs in DFSCs treated by 5 mM NAC showing top 10 ranked GO terms, including “extracellular matrix organization (GO: 0030198)” and “osteoblast differentiation (GO: 0001649)”. (F) Heatmap of DEGs involved in “extracellular matrix organization (GO: 0030198)”. (G) Heatmap of DEGs involved in “osteoblast differentiation (GO: 0001649)”. (H) Venn diagram of genes between “extracellular matrix organization (GO: 0030198)” and “osteoblast differentiation (GO: 0001649)”. Protein–protein interaction (PPI) network analysis of DEGs involved in “extracellular matrix organization (GO: 0030198)” and “osteoblast differentiation (GO: 0001649)” shows crucial nodes including COL1A1 and COL6A1. (I) Representative immunofluorescence staining of COL1A1 in DFSCs after NAC treatment. Scale bar, 25 μm. (J) Representative immunofluorescence staining of COL6A1 in DFSCs after NAC treatment. Scale bar, 25 μm. (K) Quantification of immunofluorescence staining of COL1A1 and COL6A1 in I, J. *p < 0.05 vs. control; *n* = 8 for each group. (L) Schematic diagram of COL1A1 and COL6A1 protein structure based on the Oximouse and cys‐oxidation stoichiometry. (M) Plasma levels of COL1A1 and COL6A1 after extraction of impacted mandibular third molar. *p < 0.05 vs. control; *n* = 8 for each group.

To explore the underlying mechanism, we used primary DFSCs (Figure S3) and compared the transcriptome profiles between NAC‐treated DFSCs and control cells utilizing RNA‐sequencing (SRA data: PRJNA780260). GSEA was used to identify the nature of all differential genes (*P* < 0.05) at a functional level and revealed significant enhancement of “extracellular structure organization (GO: 0043062)” and “bone development (GO: 0060348)” after NAC treatment, with the latter in particular showing the highest enrichment score of 2.34 (Figure 2A,B). A total of 685 differentially expressed genes (DEGs) were then screened based on a rigid threshold of fold change > 1.5 and *Q* < 0.01, and the distinct transcription pattern was illustrated by the heatmap (Figure 2C) and volcano map (Figure 2D). Consistent with the GSEA results, GO enrichment analysis showed that “extracellular matrix organization (GO: 0030198)” and “osteoblast differentiation (GO: 0001649)” were significantly over‐represented (Figure 2E) among 62 enriched GO terms (*Q* < 0.05) of the differential genes (Table S3), confirming that NAC played a primary role in promoting osteogenesis of DFSCs.

To gain molecular insight, gene sets of “extracellular matrix organization (GO: 0030198)” and “osteoblast differentiation (GO: 0001649)” were isolated (Figure 2F,G) and subjected to protein–protein interaction (PPI) analysis. COL1A1 and COL6A1 were significantly involved and overlapped in these two GO terms (Figure 2H) and the upregulation of COL1A1 and COL6A1 expression was validated by immunofluorescence (Figure 2I–K). Moreover, we examined whether the promoting effects on their expression were specific for NAC or could be extended to other antioxidant agents; thereby, treatments with the most common antioxidants, including NAC, N‐acetylcysteine ethyl ester (NACET), ubiquinone‐10 (CoQ10), melatonin and ascorbic acid were adopted respectively. Immunofluorescent staining and western blot showed that COL1A1 and COL6A1 were upregulated to varying degrees (Figure S4), which corroborated with the functional similarity of different antioxidants.

In general, the pro‐osteogenic effect is attributed to the regulatory role of NAC in cellular redox state. According to Oximouse (oximouse.hms.harvard.edu/index.html) [15], cys‐oxidation stoichiometry of COL6A1 showed high occupancy of cys‐oxidation at site 130 and 168 (Figure 2L), indicating that COL6A1 was more likely to be the protein target of ROS modification than COL1A1. Correspondingly, the expression of COL6A1 in plasma decreased accompanying antioxidant levels (Figure 2M). It could be speculated that redox‐sensitive COL6A1 was a potential target of NAC action in redox regulation and pro‐osteogenic effects.

### 3.3 NAC Upregulates COL6A1 Through Activating PI3K‐AKT Signalling Pathway in DFSCs

**FIGURE 3:**
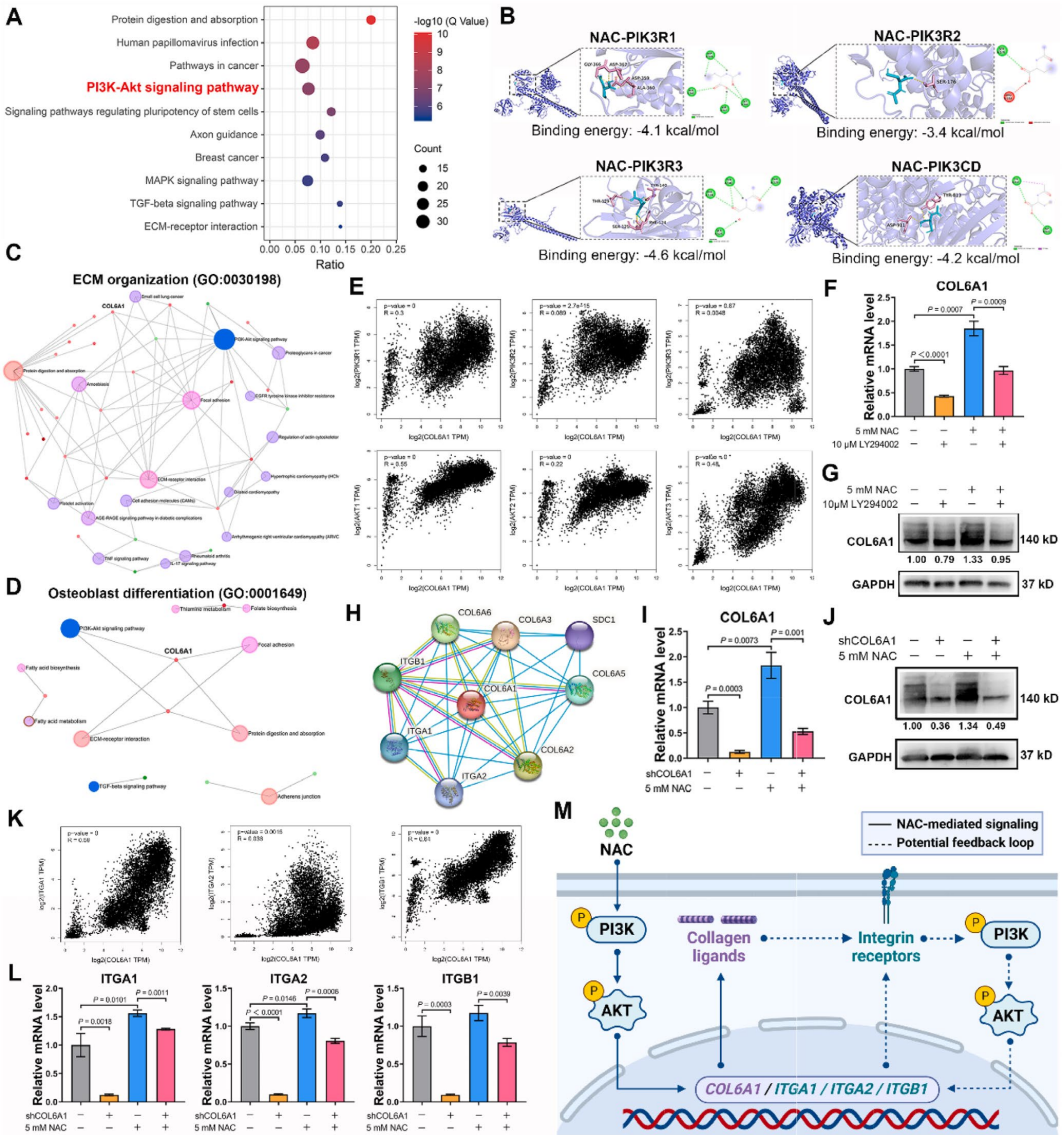
NAC upregulates COL6A1 through activating PI3K‐AKT signalling pathway in DFSCs. (A) KEGG enrichment analysis of DEGs in DFSCs after NAC treatment showing top 10 ranked KEGG pathways, including “PI3K‐AKT signaling pathway (hsa04151)”. (B) Molecular docking between NAC and PIK3RI, PIK3R2, PIK3R3 and PIK3CD by AutoDock. (https://autodock.scripps.edu/). (C) KEGG network analysis of DEGs involved in “extracellular matrix organization (GO: 0030198)” showing the association between COL6A1 and “PI3K‐AKT signaling pathway (hsa04151)”. (D) KEGG network analysis of DEGs involved in “osteoblast differentiation (GO: 0001649)” showing the association between COL6A1 and “PI3K‐AKT signaling pathway (hsa04151)”. (E) Gene correlation analysis between COL6A1 and PI3K‐AKT genes (PIK3R1, PIK3R2, PIK3R3, AKT1, AKT2, and AKT3) (http://gtexportal.org). (F) Relative mRNA levels of COL6A1 in DFSCs after NAC treatment and LY294002 treatment. *p < 0.05 vs. control; *n* = 3 independent experiments. (G) Western blot analysis of COL6A1 in DFSCs after NAC treatment and LY294002 treatment. (H) PPI physical network analysis based on STRING (string‐db.org/) between COL6A1 and integrin genes (ITGA1, ITGA2, and ITGB1). (I) Relative mRNA levels of COL6A1 in DFSCs after COL6A1 knockdown and NAC treatment. *p < 0.05 vs. control; *n* = 3 independent experiments. (J) Western blot analysis of COL6A1 in DFSCs after COL6A1 knockdown and NAC treatment. (K) Gene correlation analysis between COL6A1 and integrin genes (ITGA1, ITGA2, and ITGB1) (http://gtexportal.org). (L) Relative mRNA levels of ITGA1, ITGA2, and ITGB1 in DFSCs after COL6A1 knockdown and NAC treatment under normal condition. *p < 0.05 vs. control; *n* = 3 independent experiments. (M) Schematic diagram of the proposed mechanistic hypothesis of the positive feedback loop among COL6A1, integrins, and the PI3K‐AKT pathway triggered by NAC.

It has demonstrated that the PI3K‐AKT pathway serves as a key mechanism of redox regulation [32]. As shown in KEGG enrichment (Figure 3A), “PI3K‐Akt signaling pathway (hsa04151)” was over‐represented among 96 enriched KEGG terms (*Q* < 0.05) of the differential genes (Table S4). The AutoDock analysis further showed the binding energy between NAC and PI3K isoforms below 0 (Figure 3B), suggesting that NAC might alter signalling cascades by spontaneously binding to PI3K isoforms. To investigate whether there was a regulatory relationship between PI3K‐AKT signalling and COL6A1, gene sets of “extracellular matrix organization (GO: 0030198)” and “osteoblast differentiation (GO: 0001649)” were subjected to KEGG pathway network analysis and the results demonstrated that COL6A1 was associated with the PI3K‐AKT pathway in these two GO terms (Figure 3C,D). Similarly, gene expression correlation analysis through GTEx (gtexportal.org/) showed that COL6A1 had positive correlations with PIK3R1, AKT1, AKT2, and AKT3 (Figure 3E). We then interfered with the PI3K signals by LY294002 and measured the expression of COL6A1 in control cells and NAC‐treated cells. After treatment, RT‐qPCR and western blot revealed a significant reduction at both mRNA and protein levels (Figure 3F,G), indicating that COL6A1 was regulated by the PI3K‐AKT pathway and thus NAC activated PI3K‐AKT signalling to promote COL6A1 expression. In addition, the PPI physical network built on STRING (string‐db.org/) [33] showed high confidence interaction (score > 0.700) between COL6A1 and integrins (ITGA1, ITGA2, ITGB1) (Figure 3H). In order to explore the potential regulatory relationship, we established the COL6A1‐knockdown transfection model in DFSCs by lentiviral‐based shRNA expression and verified the knockdown efficiency utilizing RT‐qPCR (Figure 3I) and western blot (Figure 3J). COL6A1 was shown to be strongly correlated with ITGA1 and ITGB1 (Figure 3K) and RT‐qPCR further revealed that COL6A1 knockdown reduced integrin expression regardless of NAC treatment (Figure 3L), indicating that the presence of COL6A1 might be indispensable for the expression of integrins. Based on the KEGG database, ECM proteins (includes COL6A1) acted as the major ligands for integrins (includes ITGA1, ITGA2, and ITGB1) to initiate activation of PI3K‐AKT signalling. Moreover, the expression of integrins was also regulated by the PI3K‐AKT pathway according to previous studies by us and others [34, 35]. These findings support a mechanistic hypothesis of a positive feedback loop among COL6A1, integrins, and the PI3K‐AKT pathway triggered by NAC (Figure 3M).

### 3.4 COL6A1 Deficiency Disrupts Cellular Redox Balance and NAC Antioxidant Function

**FIGURE 4:**
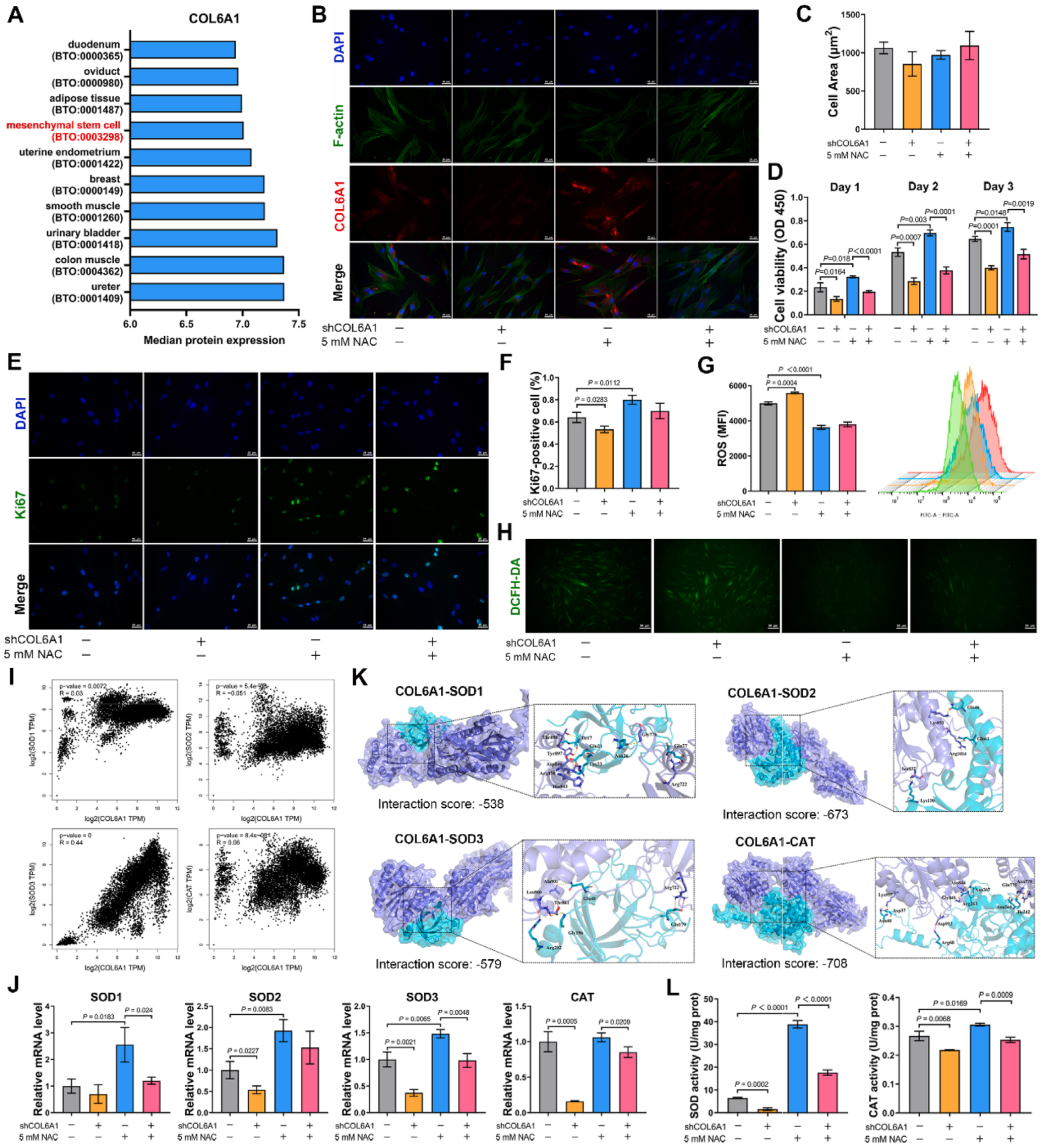
COL6A1 deficiency disrupts cellular redox balance and NAC antioxidant function. (A) Median protein expression of COL6A1 in top 10 ranked tissues or cell lines (http://www.proteomicsdb.org/). (B) Representative immunofluorescence staining of F‐actin in DFSCs after COL6A1 knockdown and NAC treatment. Scale bar, 25 μm. (C) Quantification of immunofluorescence staining indicating the cell area in B. *p < 0.05 vs. control; *n* = 3 independent experiments. (D) Cell viability analysis in DFSCs after COL6A1 knockdown and NAC treatment at day 1, 2, and 3. *p < 0.05 vs. control; *n* = 3 independent experiments. (E) Representative immunofluorescence staining of Ki67 in DFSCs after COL6A1 knockdown and NAC treatment. Scale bar, 25 μm. (F) Quantification of Ki67‐positive cell proportion in E. *p < 0.05 vs. control; *n* = 3 independent experiments. (G) Quantification of DCFH‐DA staining by Flow cytometry. *p < 0.05 vs. control; *n* = 3 independent experiments. (H) Representative images of DCFH‐DA staining in DFSCs after COL6A1 knockdown and NAC treatment. Scale bars, 50 μm. (I) Gene correlation analysis between COL6A1 and genes encoding antioxidant enzymes (SOD1, SOD2, SOD3, and CAT) (http://gtexportal.org). (J) Relative mRNA levels of SOD1, SOD2, SOD3 and CAT in DFSCs after COL6A1 knockdown and NAC treatment. *p < 0.05 vs. control; *n* = 3 independent experiments. (K) Molecular docking between COL6A1 and SOD1, SOD2, SOD3, and CAT by AutoDock. (https://autodock.scripps.edu/) (L) Quantification of SOD and CAT activity in DFSCs after COL6A1 knockdown and NAC treatment. *p < 0.05 vs. control; *n* = 3 independent experiments.

Based on STOmicsDB (db.cngb.org/stomics/) and scRNASeqDB (bioinfo.uth.edu/scrnaseqdb/), COL6A1 was distributed in all developmental stages of mouse embryos and ranked high in human embryonic stem cells, suggesting a robust association between COL6A1 and stem cell biology. ProteomicsDB (proteomicsdb.org/) further showed that the median protein expression of COL6A1 in mesenchymal stem cells ranked in the top 10 among human tissues (Figure 4A). However, the specific role of COL6A1 remains largely unknown due to previous studies which focus mostly on adult cells rather than stem cells. Firstly, we found that COL6A1 knockdown did not alter the spindle‐shaped morphology and the cell area indicated by F‐actin staining (Figure 4B,C), while cell viability (Figure 4D) clearly decreased, as well as the number of Ki67‐positive proliferating cells (Figure 4E,F). COL6A1 deficiency was reported to cause cellular oxidative stress [20], thus, we measured ROS levels using DCFH‐DA probe. Flow cytometry showed that intracellular ROS levels were remarkably upregulated with COL6A1 knockdown and decreased after NAC treatment (Figure 4G). Fluorescence microscopy revealed similar results (Figure 4H). We next examined whether COL6A1 deficiency impaired antioxidant defences. COL6A1 was found to be positively correlated with the antioxidant gene SOD3 (Figure 4I). RT‐qPCR further showed that COL6A1 knockdown was followed by downregulation of major antioxidant genes (SOD1, SOD2, SOD3, and CAT) in both control and NAC‐treated cells (Figure 4J). Moreover, the AutoDock prediction showed that COL6A1 had favourable binding scores with SOD1, SOD2, SOD3, and CAT, respectively (Figure 4K), hinting at a possible role of COL6A1 as a docking protein involved in antioxidant defences. Correspondingly, COL6A1 knockdown impaired the activities of SOD and CAT, as well as the antioxidant efficacy of NAC (Figure 4L). These data indicated that COL6A1 deficiency made the redox balance tilted toward the oxidant side.

### 3.5 COL6A1 Deficiency Impairs Mitochondrial and Autophagic Functions

**FIGURE 5:**
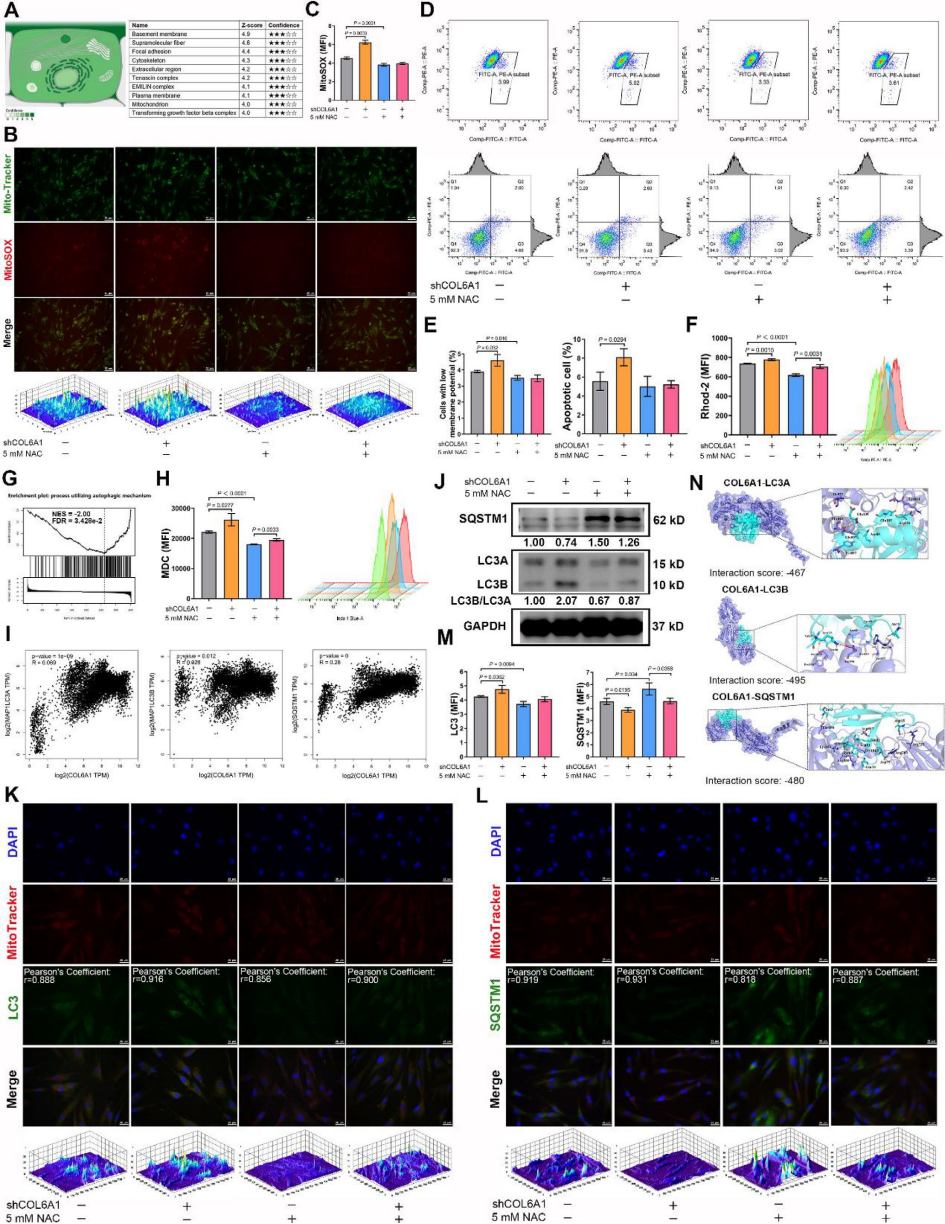
COL6A1 deficiency impairs mitochondrial and autophagic functions. (A) The association between COL6A1 and mitochondria (http://compartments.jensenlab.org/). (B) Representative fluorescent imaging depicting that MitoSox Red is colocalized with MitoTracker Green in DFSCs after COL6A1 knockdown and NAC treatment. Scale bar, 50 μm. (C) Relative MFI of MitoSox intensity detected by Image J in B. *p < 0.05 vs. control; *n* = 3 independent experiments. (D) Flow cytometry analysis of JC‐1 and Annexin V‐FITC/PI staining respectively showing mitochondrial membrane potential and apoptosis in DFSCs after COL6A1 knockdown and NAC treatment. (E) Quantification of cell proportion with low mitochondrial membrane potential and apoptotic cell ratio. *p < 0.05 vs. control; *n* = 3 independent experiments. (F) Quantification of Rhod‐2 staining by Flow cytometry showing mitochondrial calcium ion in DFSCs after COL6A1 knockdown and NAC treatment. *p < 0.05 vs. control; *n* = 3 independent experiments. (G) GSEA showing decreased enrichment of autophagy‐related genes in DFSCs after NAC treatment. (H) Quantification of MDC staining by Flow cytometry in DFSCs after COL6A1 knockdown and NAC treatment. *p < 0.05 vs. control; *n* = 3 independent experiments. (I) Gene correlation analysis between COL6A1 and LC3A, LC3B, and SQSTM1 (http://gtexportal.org). (J) Western blot analysis of LC3A, LC3B, and SQSTM1 in DFSCs after COL6A1 knockdown and NAC treatment. (K) Representative immunofluorescence staining of LC3 in DFSCs after COL6A1 knockdown and NAC treatment. Scale bar, 25 μm. (L) Representative immunofluorescence staining of SQSTM1 in DFSCs after COL6A1 knockdown and NAC treatment. Scale bar, 25 μm. (M) Quantification of immunofluorescence staining of LC3 and SQSTM1 in K, L. *p < 0.05 vs. control; *n* = 3 independent experiments. (N) Molecular docking between COL6A1 and LC3A, LC3B, and SQSTM1 by AutoDock. (https://autodock.scripps.edu/).

Mitochondria were both major sources and targets of ROS [36] while the clues about mitochondrial dysfunction and apoptosis were uncovered by ultrastructural analysis in COL6A1 knockout mouse muscle [37, 38]. COMPARTMENTS (compartments.jensenlab.org/) also revealed the association between COL6A1 and mitochondria (Figure 5A). Therefore, we examined several indicators of mitochondrial redox state. MitoSOX fluorescence showed an increase in mitochondrial ROS levels after COL6A1 knockdown (Figure 5B,C). Flow cytometry further demonstrated that COL6A1 knockdown led to an elevation in the number of cells with low mitochondrial membrane potential (Figure 5D) and an increase in apoptotic ratio (Figure 5E). Excessive uptake of calcium ions is known to induce mitochondrial oxidative stress [39]. Not surprisingly, the mitochondrial calcium content increased in COL6A1‐knockdown cells as well (Figure 5F). These results suggested that COL6A1 was involved in the regulation of mitochondrial redox state. Yet, it could be witnessed that the improved mitochondrial status under NAC treatment was not much altered due to COL6A1 deficiency.

Autophagy is vital for intracellular homeostasis. Typically, the autophagic activity is greatly increased under oxidative stress and can be rescued by antioxidants [40, 41]. According to the above sequencing results, GSEA revealed a negative correlation between NAC and “process utilizing autophagic mechanism (GO: 0061919)” (Figure 5G). Flow cytometry of fluorescent probe MDC, a marker for autophagolysosomes, confirmed this point and further showed a significant enhancement of autophagy under COL6A1 deficiency (Figure 5H). This prompted us to investigate the relationship of COL6A1 with key autophagy markers, LC3 (LC3A and LC3B) and SQSTM1. COL6A1 was found to be correlated with SQSTM1 instead of LC3 at the gene level (Figure 5I), whereas western blot analysis revealed that the ratio of LC3B/LC3A was upregulated and the expression of SQSTM1 was decreased following COL6A1 knockdown at the protein level (Figure 5J). Meanwhile, these expression alterations in NAC‐treated cells were opposite to the trend under COL6A1 knockdown (Figure 5J), suggesting that excessive autophagy activated by COL6A1 deficiency could be rescued by NAC. Immunofluorescence staining results were consistent with the western blot (Figure 5K–M). Furthermore, fluorescence colocalization analysis revealed that both LC3 and SQSTM1 had strong colocalization with Mito Tracker (colocalization coefficient > 0.8), indicating a possible involvement of mitophagy (Figure 5K,L). Finally, molecular docking analysis showed good binding affinities between COL6A1 and these autophagy markers at the molecular level (Figure 5N), which provided additional evidence for a direct role of COL6A1 in the regulation of autophagy in stem cells. Taken together, COL6A1 participated in the maintenance of cellular redox homeostasis by coordinating mitochondrial and autophagic functions.

### 3.6 COL6A1 Ensures Normal Osteogenesis and Enhances NAC'S Pro‐Osteogenic Activity

**FIGURE 6:**
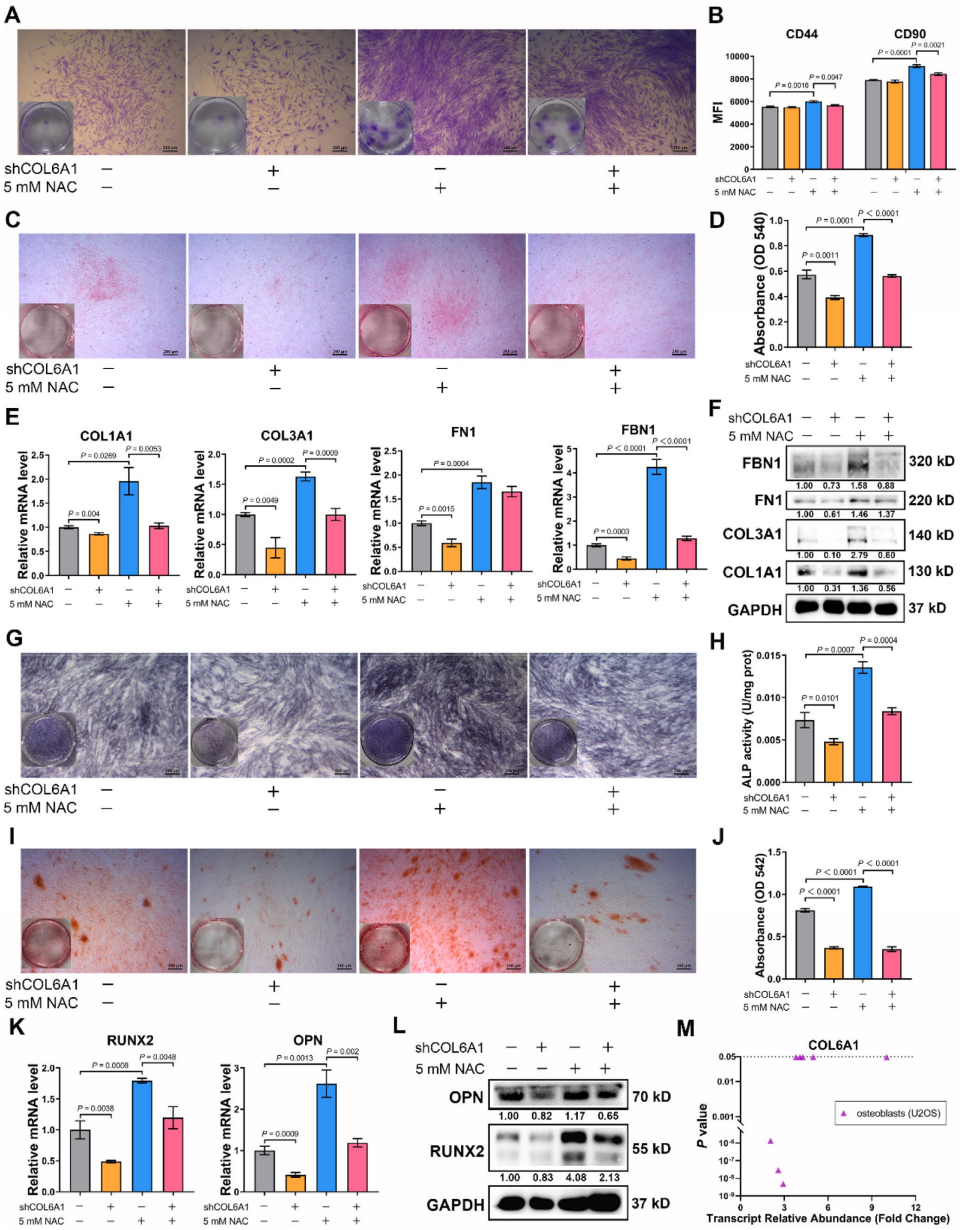
COL6A1 ensures normal osteogenesis and enhances NAC's pro‐osteogenic activity. (A) Representative images of colony formation assay in DFSCs after COL6A1 knockdown and NAC treatment. Scale bars, 250 μm. (B) Flow cytometry analysis of the expression of stemness markers CD44 and CD90 in DFSCs after COL6A1 knockdown and NAC treatment. *p < 0.05 vs. control; *n* = 3 independent experiments. (C) Representative images of sirius red staining showing ECM production in DFSCs after COL6A1 knockdown and NAC treatment under normal conditions. Scale bars, 250 μm. (D) Quantification of sirius red staining in C. *p < 0.05 vs. control; *n* = 3 independent experiments. (E) Relative mRNA levels of COL1A1, COL3A1, FN1 and FBN1 in DFSCs after COL6A1 knockdown and NAC treatment under normal condition. *p < 0.05 vs. control; *n* = 3 independent experiments. (F) Western blot analysis of COL1A1, COL3A1, FN1, and FBN1 in DFSCs after COL6A1 knockdown and NAC treatment under normal condition. (G) Representative images of ALP staining showing osteogenic differentiation in DFSCs after COL6A1 knockdown and NAC treatment under osteogenic induction. Scale bars, 250 μm. (H) Quantification of ALP activity in G. *p < 0.05 vs. control; *n* = 3 independent experiments. (I) Representative images of ARS staining showing osteogenic maturation in DFSCs after COL6A1 knockdown and NAC treatment under osteogenic induction. Scale bars, 250 μm. (J) Quantification of ARS staining in I. *p < 0.05 vs. control; *n* = 3 independent experiments. (K) Relative mRNA levels of RUNX2 and OPN in DFSCs after COL6A1 knockdown and NAC treatment under osteogenic induction. *p < 0.05 vs. control; *n* = 3 independent experiments. (L) Western blot analysis of RUNX2 and OPN in DFSCs after COL6A1 knockdown and NAC treatment under osteogenic induction. (M) Relative transcript abundance of COL6A1 in osteoblast cell line (http://www.signalingpathways.org).

An imbalance of the redox equilibrium commonly impairs the functional integrity of stem cells. We therefore examined whether COL6A1 deficiency altered the regenerative abilities of DFSCs and the promoting effects of NAC. Although COL6A1 knockdown did not have a significant effect on the clone formation ability, it weakened the effect of NAC (Figure 6A). The relative expression of stemness markers CD44 and CD90 measured by flow cytometry showed similar trends (Figure 6B). There were few studies of this gene in the field of bone biology at present, but we found some clues obtained from public databases. Based on experiments from TISSUES (tissues.jensenlab.org/), the relationship between COL6A1 and bone ranked second among human tissues (Table S5). Meanwhile, MalaCards (malacards.org/) revealed that COL6A1 was associated with several bone diseases such as hyperostosis and diffuse idiopathic skeletal hyperostosis (Table S6). According to the primary cell atlas dataset from bioGPS (biogps.org/), COL6A1 was more highly expressed in mesenchymal stem cells and was upregulated with the extension of osteogenic induction time. Therefore, it was inferred that COL6A1 might be closely associated with osteogenic differentiation. It has been studied that orchestration of ECM and osteogenic gene expression is indispensable for proper execution of osteogenesis [18]. We found that COL6A1 had significant correlations with the ECM genes (COL1A1, COL3A1) (Figure S5A) and the osteogenic factor (RUNX2) (Figure S5B). RT‐qPCR confirmed that COL6A1 expression increased in a time‐dependent manner during the natural deposition of ECM (Figure S5C) and the osteogenic induction (Figure S5D), which suggested the potential role of COL6A1 in “extracellular matrix organization (GO: 0030198)” and “osteoblast differentiation (GO: 0001649)”. Then, analysis of picro‐sirius red staining showed that COL6A1 knockdown reduced ECM deposition in control cells and NAC‐treated cells (Figure 6C,D). The expression levels of typical ECM components, including COL1A1, COL3A1, FN1 and FBN1, were correspondingly changed by RT‐qPCR and western blot (Figure 6E,F) which were consistent with RNA‐sequencing data. ALP and ARS activity assays further showed that COL6A1 knockdown not only disrupted normal osteogenic differentiation of DFSCs but also offset the facilitation of NAC (Figure 6G–J). The expression levels of osteogenic markers, RUNX2 and OPN exhibited a similar tendency (Figure 6K,L). Data from Signalling Pathways Project (signalingpathways.org/) also corroborated the high expression of COL6A1 in the osteoblast cell line (Figure 6M). Thus, our results demonstrated that COL6A1 played an important role in the osteogenic process of DFSCs and was involved in the biological functions of NAC.

### 3.7 NAC Treatment in DFSCs Enhances Bone Healing via COL6A1 Upregulation

**FIGURE 7:**
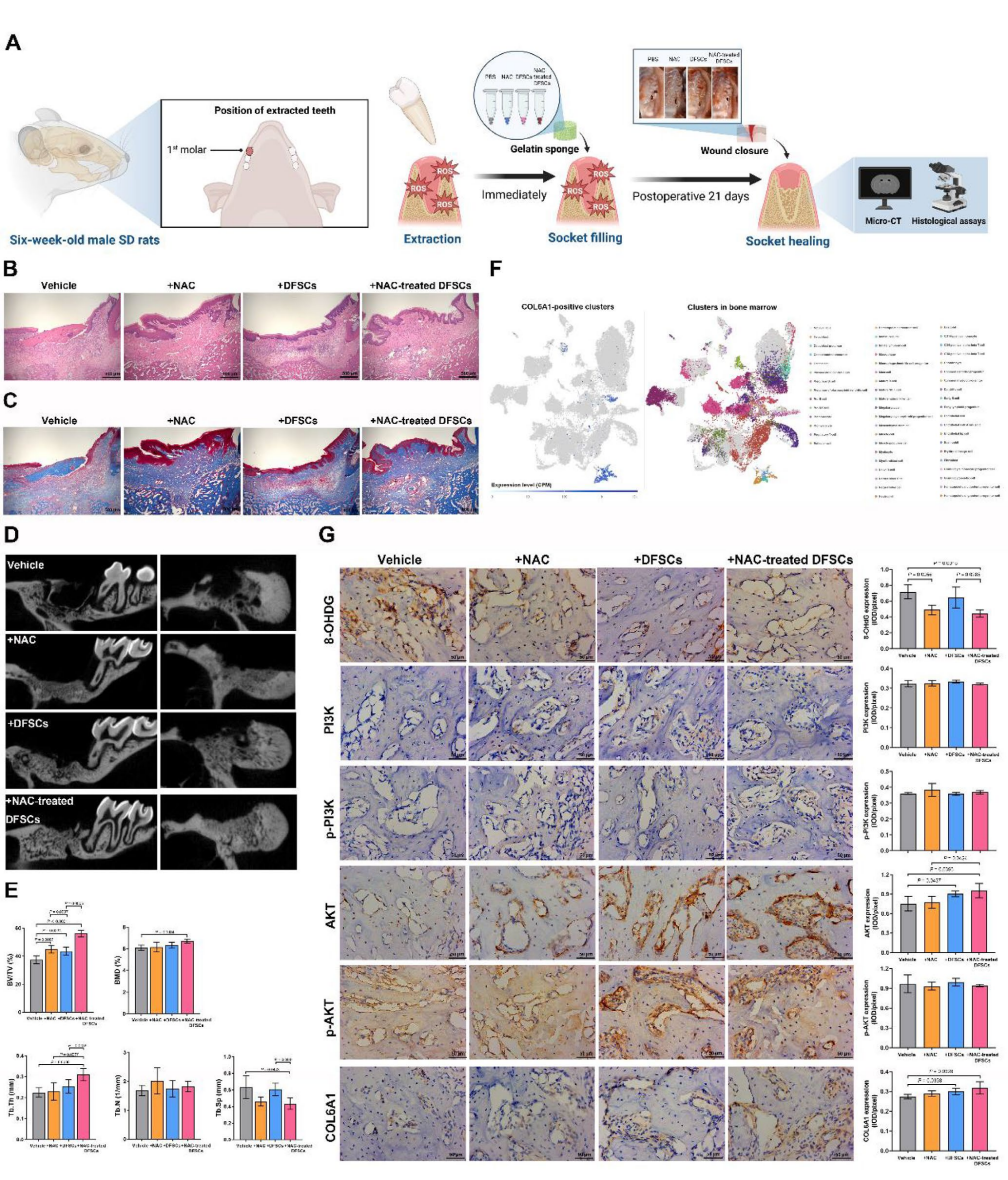
NAC treatment in DFSCs enhances bone healing via COL6A1 upregulation. (A) Schematic diagram of the animal experiments. (B) Representative images of HE staining in extraction sockets. Scale bar, 500 μm. (C) Representative images of Masson's trichrome staining in extraction sockets. Scale bar, 500 μm. (D) Representative images of micro‐CT reconstruction and three‐dimensional section at 3 weeks after tooth extraction. (E) Quantitative micro‐CT analysis of bone volume/total volume (BV/TV), bone mineral density (BMD), trabecular thickness (Tb.Th), trabecular number (Tb.N), and trabecular separation (Tb.Sp). *p < 0.05 vs. control; *n* = 4 for each group. (F) The predominant expression of COL6A1 in mesenchymal stem cells and osteoblast precursors (ebi.ac.uk/gxa/sc/) (G) Representative immunohistochemistry staining and statistical analysis of key signalling molecules (PI3K, phosphorylated PI3K, AKT, phosphorylated AKT) and oxidative stress marker (8‐OHdG) in rats after treatment for 3 weeks. Scale bar, 50 μm. *p < 0.05 vs. control; *n* = 4 for each group.

Rat DFSCs were identified (Figure S6) and loaded onto gelatin sponge scaffold for in vivo transplantation. We also found that the wound of alveolar sockets was nearly closed in four groups at day 21. Combined with others' findings [42, 43, 44], histological examination was performed at this time point for evaluation of bone healing (Figure 7A). HE staining showed grossly normal tissue structures without obvious adverse reactions such as infection or necrosis (Figure 7B). Masson trichrome staining showed that ECM deposition appeared prevalent in new tissues of alveolar sockets (Figure 7C). We then evaluated the osteogenesis within the socket region of interest. Micro‐CT revealed that more newly formed osteoid tissues were observed in the sites filled with NAC‐treated DFSCs than in other groups while NAC or DFSCs alone also increased osteoid formation compared to the control (Figure 7D). This pattern was similarly reflected in the micro‐CT quantification. Compared to the control, BV/TV remarkably increased after local transplantation of NAC, DFSCs, or NAC‐treated DFSCs, with the highest potency of the latter which exhibited an elevation of 18.7% (Figure 7E). There was a slight rise in BMD only in the treatment group with NAC‐treated cells (Figure 7E). Concerning trabecular microarchitecture, treatment of stem cells with NAC showed increased trabecular thickness and reduced trabecular separation, while trabecular number was unchanged (Figure 7E).

According to single cell gene profiling of human bone marrow from Single Cell Expression Atlas (ebi.ac.uk/gxa/sc/), COL6A1 was mainly expressed in mesenchymal stem cells and osteoblast precursors (Figure 7F), indicating that COL6A1 functioned primarily in stem cell‐mediated osteogenesis. In agreement with in vitro results, transplantation of stem cells or NAC‐treated cells exhibited upregulation of COL6A1 as revealed by immunohistochemistry (Figure 7G). Although immunohistochemical staining for 8‐OHdG verified the inhibition of oxidative stress in groups treated with NAC or NAC‐treated cells, no significant activation of the PI3K‐AKT pathway was observed (Figure 7G), probably because sustained signalling was not required for tissue repair except for tumours with activating mutations in PI3K. Collectively, NAC conferred superior bone healing properties to stem cell‐based therapy via upregulation of COL6A1.

## 4 Discussion

Stem cells are sensitive to ROS, making ROS‐driven oxidative stress crucial in stem cell therapy. Antioxidant treatments, like NAC, have been explored to boost stem cell efficacy. While NAC is known to protect stem cells from oxidative stress, its critical signalling axis in treating alveolar bone injury is unclear [10, 45, 46]. Our study shows that NAC enhances antioxidative and pro‐osteogenic effects in stem cell therapy by activating the PI3K‐AKT‐COL6A1 axis, potentially offering a new target for antioxidant treatments in regenerative medicine. 

To investigate the underlying molecular mechanisms, we administered antioxidants to DFSCs and conducted a comparative analysis of transcriptional data between NAC‐treated and untreated DFSCs. Our findings suggest that NAC's enhancement of osteogenesis is associated with the key genes COL1A1 and COL6A1, corroborating previous research that highlights their significant role in osteogenic processes [47, 48]. Utilizing public databases for cysteine oxidation assessment, COL6A1 was finally identified as a potential target for ROS modification in current research. Studies on COL6A1^−/−^ mice have shown increased ROS levels, which suggest a strong association between COL6A1 and oxidative stress [21, 37, 38]. Our KEGG analysis and PI3K inhibition assay further implied that there was a regulatory relationship between COL6A1 and PI3K‐AKT in the bone‐promoting effect of NAC. COL6A1 is known to interact with a range of cell surface receptors, such as integrins, which are required for PI3K activation [20, 49]. We unexpectedly discovered that COL6A1 deficiency down‐regulated the expression of integrins, indicating a potential upstream regulator of COL6A1 for the PI3K‐AKT pathway. These results propose a conjecture of a positive regulatory loop among COL6A1, integrins, and the PI3K‐AKT signalling pathway, which will be considered in our future studies.

As COL6A1 is a potential redox regulator downstream of antioxidants, we investigated its role in redox homeostasis and its impact on osteogenesis. Our findings revealed that COL6A1 deficiency not only elevated ROS levels but also disrupted antioxidant enzyme systems, resulting in oxidative stress. Redox regulation is multifaceted, involving oxidation–reduction reactions, mitochondrial functions, and autophagy [5, 32, 50]. Mitochondria, as the central hub for intracellular ROS management, are particularly relevant. Previous studies have linked COL6A1 genomic deletions to mitochondrial defects in Bethlem myopathy and Ullrich muscular dystrophy, manifesting as increased mitochondrial ROS, membrane destabilization, and apoptosis [20, 51]. Consistent with these reports, our study demonstrated that COL6A1 knockdown upregulated mitochondrial ROS levels, induced MMP loss, and altered mitochondrial calcium uptake, suggesting a critical role of COL6A1 in maintaining mitochondrial redox balance in stem cells. Notably, recent significant research highlighted the extracellular matrix (ECM) as a regulator of mitochondrial homeostasis [52]. Our work extends this understanding by showing that COL6A1, a microfilament protein, also contributes to the positive regulation of ECM.

Autophagy is another important biological process for stem cell function, as it preserves stemness and quiescence by eliminating damaged mitochondria and organelles [53]. Excessive ROS triggers autophagy as a negative feedback mechanism to restore redox homeostasis. Autophagy dynamically adjusts its basal state in a cell type‐dependent manner to maintain redox balance [54]. While COL6A1 has been reported to modulate autophagy variably across cell types [49], our study found that COL6A1 deficiency led to autophagosome accumulation and altered expression of autophagy‐related proteins. This response aligns with findings in fibroblasts [55] but contrasts with defective autophagy induction in muscle cells [38] and neural cells [56]. Given the observed oxidative stress, we propose that autophagy activation in this context represents a cytoprotective mechanism to counteract redox imbalance. Additionally, colocalization of LC3 and SQSTM1 with Mito Tracker also suggests the involvement of mitophagy, warranting further investigation.

This study also had some limitations. First, COL6A1 knockdown only partially impaired stem cell functions, likely due to compensatory mechanisms involving other collagen VI family members. Second, the proposed COL6A1‐integrin‐PI3K‐AKT feedback loop requires further mechanistic investigation and in vivo therapeutic verification. Third, in vivo experiments were limited to early and mid‐stage healing without long‐term assessment. These limitations will be addressed in our follow‐up studies.

## 5 Conclusion

**FIGURE 8:**
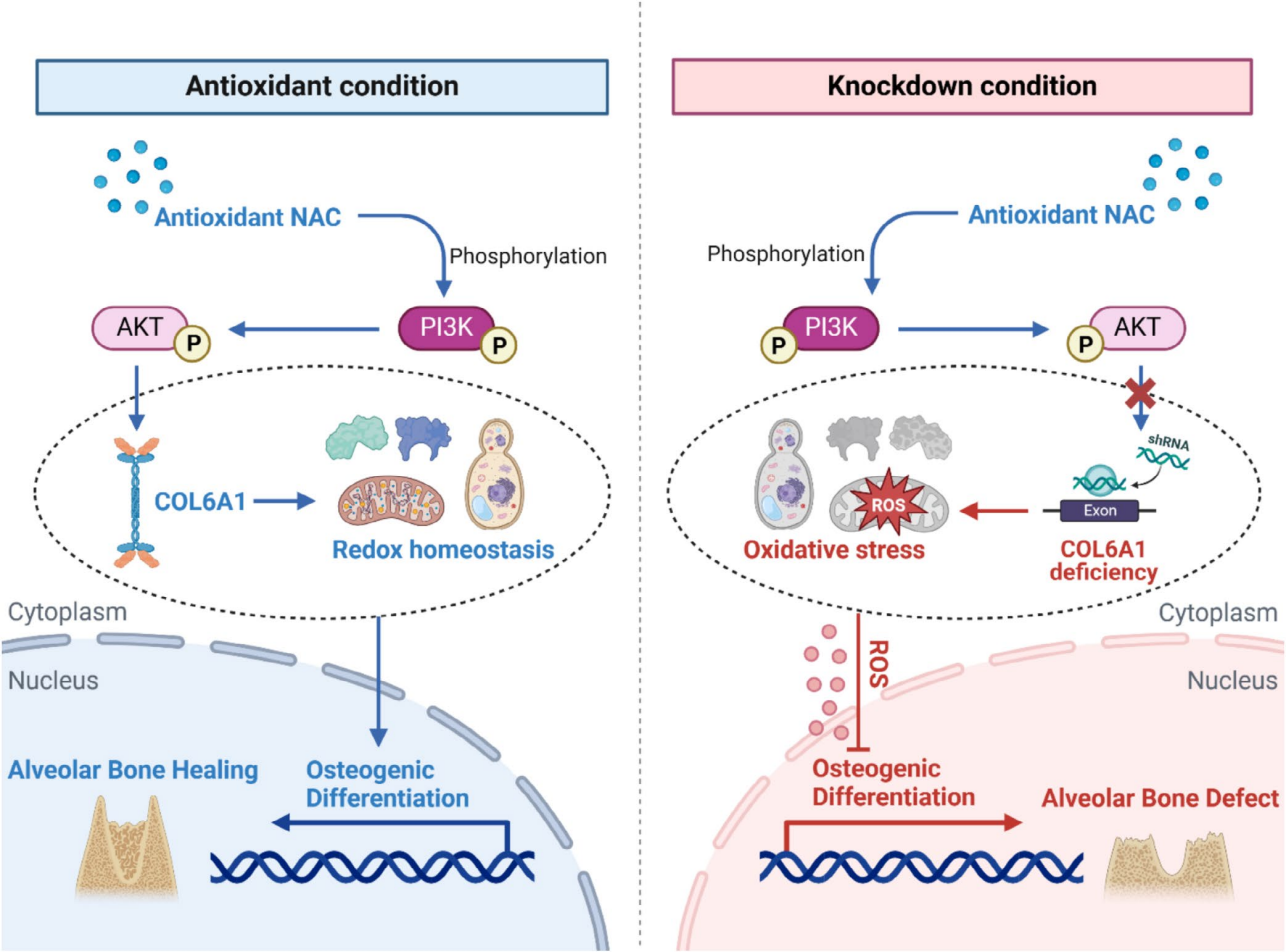
Summary of the role of COL6A1 in NAC‐promoted osteogenic effects. Under physiological conditions, NAC activates the PI3K‐AKT‐COL6A1 axis to sustain redox balance and enhance osteogenesis. COL6A1 deficiency, however, compromises this axis, inducing oxidative stress and impairing NAC's osteogenic efficacy.

In conclusion, our findings establish COL6A1 as a key downstream effector of antioxidant responses that maintains redox homeostasis and promotes osteogenesis in stem cell‐based therapies (Figure 8). While challenges remain in protocol standardization and long‐term validation, the NAC‐COL6A1 axis offers significant translational potential for bone regeneration. Further elucidation of the COL6A1‐integrin‐PI3K/AKT feedback loop may yield novel strategies to optimize regenerative outcomes, particularly in dental and maxillofacial applications.

## Author Contributions

Conceptualization: Z.M., J.L., Y.Z., L.H., L.S. Methodology: J.L, Y.Z., R.Y. Investigation: Z.M., J.L., Y.Z., R.Y., J.Z., H.Y., Z.W., R.W., Z.L., S.G., Visualization: J.L., Y.Z., Z.W. Supervision: Z.M., L.H., L.S. Writing – original draft: Z.M., J.L., Y.Z. Writing – review and editing: L.H., L.S. All authors read and approved the final version of the manuscript.

## Funding

This work was supported by the National Natural Science Foundation of China, 82471026, 82301030, Tianjin Medical University 123 clinical promotion project, 0203, National Key Research and Development Program of China, 2022YFC2405901, Tianjin Health Research Project, TJWJ2024MS009, Key Program of Tianjin Natural Science Foundation, 24JCZDJC00920.

## Conflicts of Interest

The authors declare no conflicts of interest.

## Supporting information


**Figure S1:** (A) Metabolites in human plasma after extraction of impacted mandibular third molar was classified by HMDB database.
(B) Metabolites in human plasma after extraction of impacted mandibular third molar was classified by KEGG database.
(C) Heat map of differentially expressed metabolites (DEM) levels in human plasma after extraction of impacted mandibular third molar.
(D) Volcano plot of DEM levels in human plasma after extraction of impacted mandibular third molar.
(E) Bubble plots showing the expression correlation between DEM with least P value and oxidative stress.
(F) Representative violin plots of DEM distribution in E.
(G) Matrix plots showing the correlation among metabolites in E.
(H) RaMP enrichment analysis of DEM levels in human plasma after extraction of impacted mandibular third molar.
(I) KEGG enrichment analysis of DEM levels in human plasma after extraction of impacted mandibular third molar.
(J) SMPD enrichment analysis of DEM levels in human plasma after extraction of impacted mandibular third molar.
**Figure S2:** (A) Lipid metabolites in human plasma after extraction of impacted mandibular third molar was classified by Lipidmaps database.
(B) Heat map of differentially expressed lipid metabolites levels in human plasma after extraction of impacted mandibular third molar.
(C) Volcano plot of differentially expressed lipid metabolites levels in human plasma after extraction of impacted mandibular third molar.
(D) Histogram showing the proportion of differentially expressed lipid metabolites in Fig1I‐J.
(E) Bubble plots showing the proportion of differentially expressed lipid metabolites with least *P* value.
(F) Representative violin plots of differentially expressed lipid metabolites distribution in E.
(G) Matrix plots showing the correlation among metabolites in E.
(H) RaMP enrichment analysis of differentially expressed lipid metabolites levels in human plasma after extraction of impacted mandibular third molar.
**Figure S3:** Characterization of human dental follicle stem cells (hDFSCs).
(A) Flow cytometry gating strategy. Positive markers including CD31, CD117. Negative markers involve CD29, CD44, CD90.
(B) Representative fluorescent imaging depicting that hDFSCs were stained for the mesenchymal marker (positive for Vimentin; green) and nuclei (DAPI; blue). Scale bar, 50 μm.
(C) Representative fluorescent imaging depicting that hDFSCs were stained for the epithelial marker (negative for CK14; green) and nuclei (DAPI; blue). Scale bar, 50 μm.
(D) Representative images of alkaline phosphatase staining for osteogenic differentiation after osteogenic culturing for 5 days. Scale bars: 250 μm.
(E) Representative images of alizarin red s staining for matrix mineralization after osteogenic culturing for 15 days. Scale bars, 200 μm.
(F) Representative images of oil red o staining for adipogenic differentiation after adipogenic induction for 15 days. Scale bars, 100 μm.
(G) Representative fluorescent imaging depicting that hDFSCs with neurogenic differentiation potential were stained for the neurogenic marker (positive for β‐III‐tubulin; red) and nuclei (DAPI; blue). Scale bar, 50 μm.
**Figure S4:** (A) Representative immunofluorescence staining of COL1A1 and COL6A1 in DFSCs after NAC, NACET, CoQ10, Melatonin or Ascorbic acid treatment. Scale bar, 25 μm.
(B) Quantification of immunofluorescence staining of COL1A1 and COL6A1 in A. *p < 0.05 vs. control; *n* = 3 independent experiments.
(C) Western blot analysis of COL1A1 and COL6A1 in DFSCs after NAC, NACET, CoQ10, Melatonin or Ascorbic acid treatment.
**Figure S5:** (A) Gene correlation analysis between COL6A1 and COL3A1 (http://gtexportal.org).
(B) Gene correlation analysis between COL6A1 and RUNX2, SPP1 (http://gtexportal.org).
(C) Relative mRNA levels of COL6A1 in DFSCs after ECM deposition for 0, 1, 4, 14 days. *p < 0.05 vs. control; *n* = 3 independent experiments.
(D) Relative mRNA levels of COL6A1 in DFSCs after osteogenic culturing for 0, 1, 4, 14 days. *p < 0.05 vs. control; *n* = 3 independent experiments.
**Figure S6:** Characterization of rat dental follicle stem cells (rDFSCs).
(A) Flow cytometry gating strategy. Positive markers including CD29, CD90. Negative markers involve CD11, CD45, CD106.
(B) Representative fluorescent imaging depicting that rDFSCs were stained for the mesenchymal marker (positive for Vimentin; green) and nuclei (DAPI; blue). Scale bar, 100 μm.
(C) Representative fluorescent imaging depicting that rDFSCs were stained for the epithelial marker (negative for CK14; green) and nuclei (DAPI; blue). Scale bar, 100 μm.
(D) Representative images of alkaline phosphatase staining for osteogenic differentiation after osteogenic culturing for 5 days. Scale bars, 250 μm.
(E) Representative images of alizarin red s staining for matrix mineralization after osteogenic culturing for 15 days. Scale bars, 100 μm.
(F) Representative images of oil red o staining for adipogenic differentiation after adipogenic induction for 15 days. Scale bars, 50 μm.
(G) Representative fluorescent imaging depicting that rDFSCs with neurogenic differentiation potential were stained for the neurogenic marker (positive for β‐III‐tubulin; red) and nuclei (DAPI; blue). Scale bar, 50 μm.
**Table S1:** Antioxidant treatments.
**Table S2:** Primer sequences.
**Table S3:** GO enriched terms of biological process.
**Table S4:** KEGG enriched pathways.
**Table S5:** The top 10 associations between COL6A1 and human tissues based on experiments from TISSUES (http://tissues.jensenlab.org/).
**Table S6:** The top 20 associations between COL6A1 and human diseases based on MalaCards (http://www.malacards.org/).

## Data Availability

The data for this study are available upon reasonable request to the corresponding author. The expanded data are shown in supplemental material. SRA records of RNA‐sequencing data are accessible (http://www.ncbi.nlm.nih.gov/sra/PRJNA780260). The uncropped western blot images and replicates are shown in Figureshare (DOI: 10.6084/m9.figureshare.22632625).
