# Supplementary material for: Collagen Type VI Alpha 1 as a Regulator of Redox Homeostasis in Antioxidant‐Enhanced Osteogenesis of Dental Stem Cells
Source: Cell Prolif. 2026 May 7:e70220. Online ahead of print. doi: 10.1111/cpr.70220 (PMC13325639; doi:10.1111/cpr.70220)
Supplement: Supplementary file 1 — Figure S1: (A) Metabolites in human plasma after extraction of impacted mandibular third molar was classified by HMDB database. (B) Metabolites in human plasma after extraction of impacted mandibular third molar was classified by KEGG database. (C) Heat map of differentially expressed metabolites (DEM) levels in human plasma after extraction of impacted mandibular third molar. (D) Volcano plot of DEM levels in human plasma after extraction of impacted mandibular third molar. (E) Bubble plots showing the expression correlation between DEM with least P value and oxidative stress. (F) Representative violin plots of DEM distribution in E. (G) Matrix plots showing the correlation among metabolites in E. (H) RaMP enrichment analysis of DEM levels in human plasma after extraction of impacted mandibular third molar. (I) KEGG enrichment analysis of DEM levels in human plasma after extraction of impacted mandibular third molar. (J) SMPD enrichment analysis of DEM levels in human plasma after extraction of impacted mandibular third molar. Figure S2: (A) Lipid metabolites in human plasma after extraction of impacted mandibular third molar was classified by Lipidmaps database. (B) Heat map of differentially expressed lipid metabolites levels in human plasma after extraction of impacted mandibular third molar. (C) Volcano plot of differentially expressed lipid metabolites levels in human plasma after extraction of impacted mandibular third molar. (D) Histogram showing the proportion of differentially expressed lipid metabolites in Fig1I‐J. (E) Bubble plots showing the proportion of differentially expressed lipid metabolites with least P value. (F) Representative violin plots of differentially expressed lipid metabolites distribution in E. (G) Matrix plots showing the correlation among metabolites in E. (H) RaMP enrichment analysis of differentially expressed lipid metabolites levels in human plasma after extraction of impacted mandibular third molar. Figure S3: Characterization o [file CPR-9999-e70220-s001.docx]

**Supplementary Materials for**

**• COL6A1 as a regulator of redox homeostasis in antioxidant-enhanced osteogenesis of dental stem cells**

Zhaosong Meng *et al.*

* Lei Sui. Email: suilei@tmu.edu.cn

**This file includes:**

Supplementary Figs. S1 to S6

Supplementary Tab. S1 to S6

Supplementary Fig. S1.


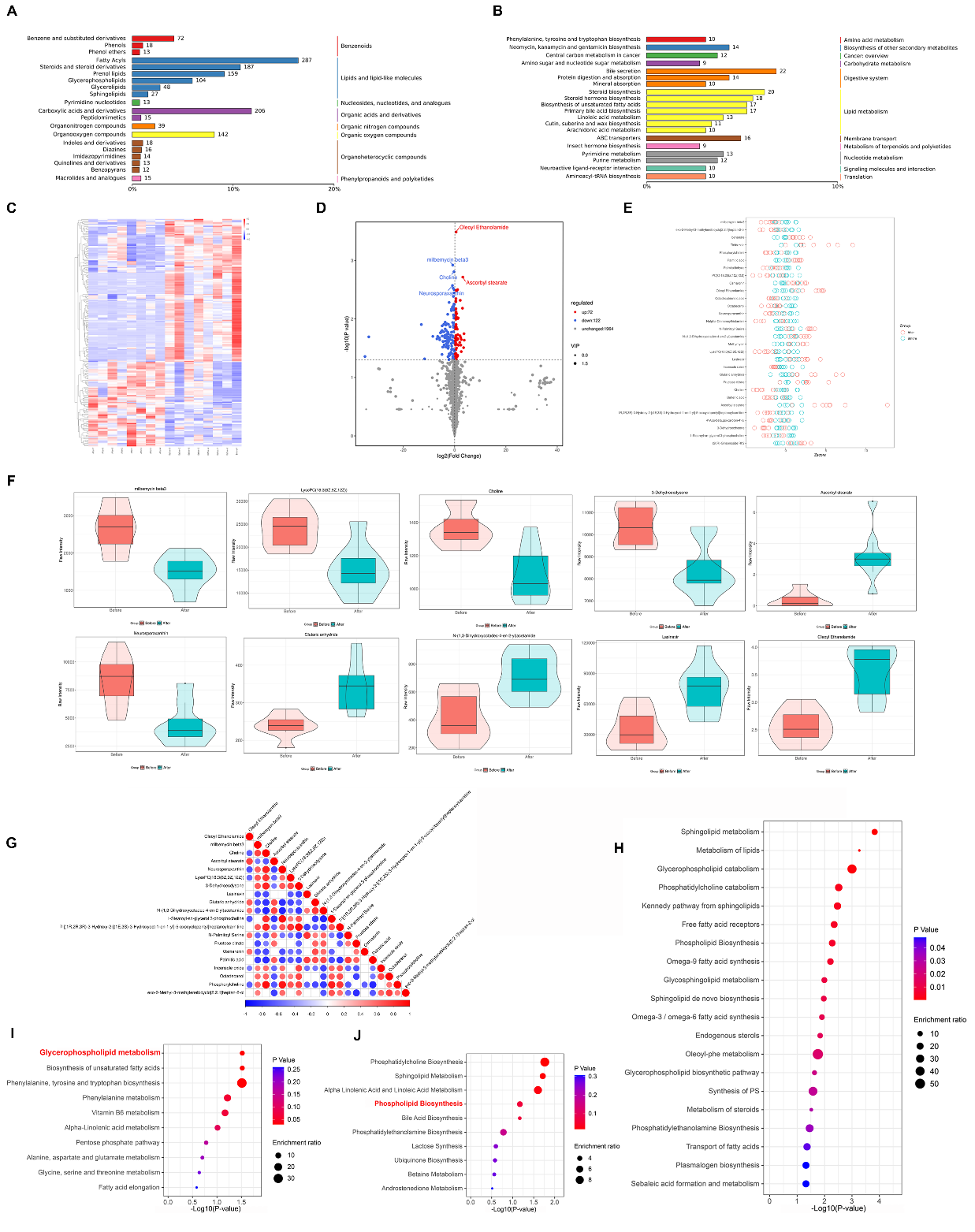


(A) Metabolites in human plasma after extraction of impacted mandibular third molar was classified by HMDB database.

(B) Metabolites in human plasma after extraction of impacted mandibular third molar was classified by KEGG database.

(C) Heat map of differentially expressed metabolites (DEM) levels in human plasma after extraction of impacted mandibular third molar.

(D) Volcano plot of DEM levels in human plasma after extraction of impacted mandibular third molar.

(E) Bubble plots showing the expression correlation between DEM with least P value and oxidative stress.

(F) Representative violin plots of DEM distribution in E.

(G) Matrix plots showing the correlation among metabolites in E.

(H) RaMP enrichment analysis of DEM levels in human plasma after extraction of impacted mandibular third molar.

(I) KEGG enrichment analysis of DEM levels in human plasma after extraction of impacted mandibular third molar.

(J) SMPD enrichment analysis of DEM levels in human plasma after extraction of impacted mandibular third molar.

Supplementary Fig. S2.


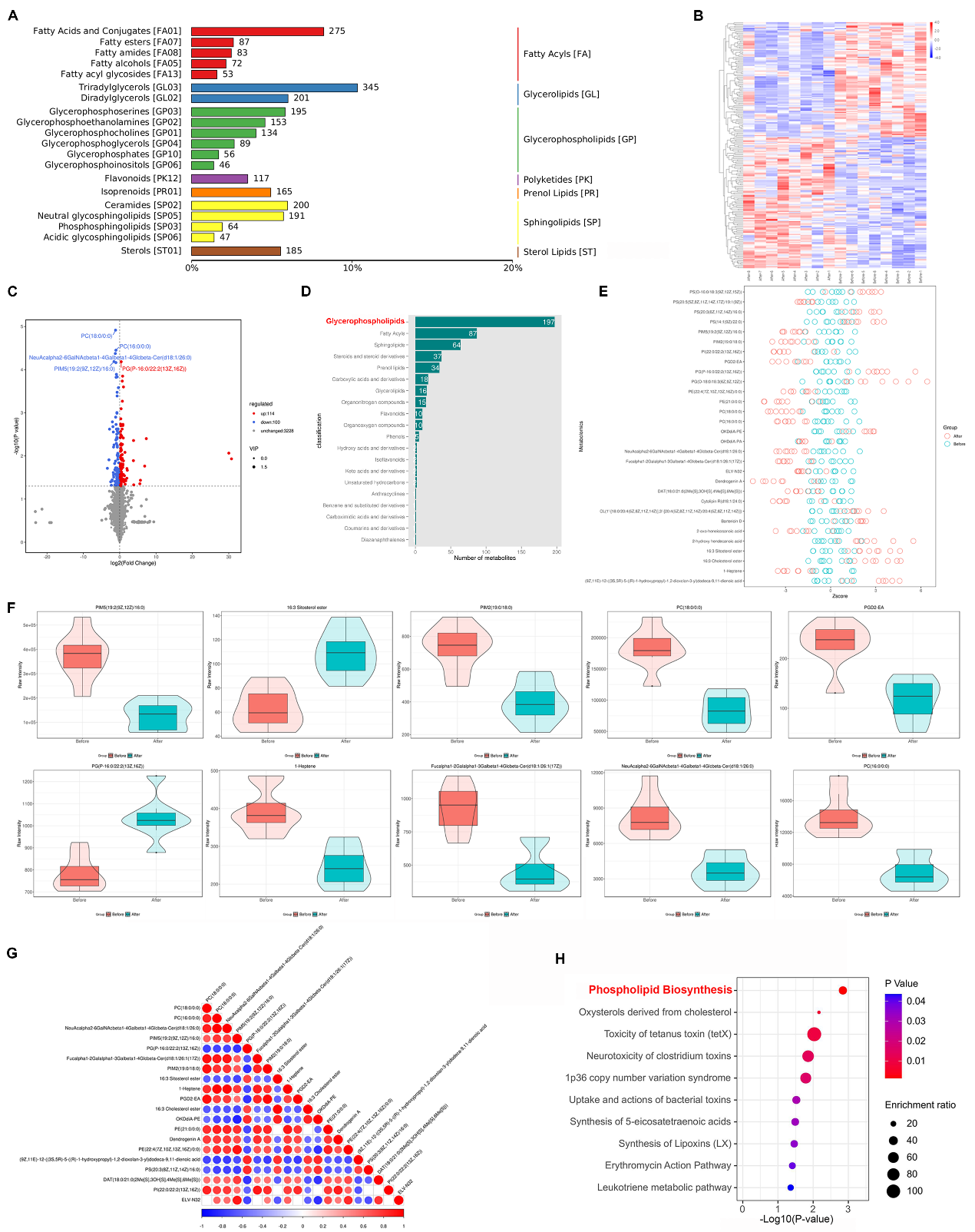


(A) Lipid metabolites in human plasma after extraction of impacted mandibular third molar was classified by Lipidmaps database.

(B) Heat map of differentially expressed lipid metabolites levels in human plasma after extraction of impacted mandibular third molar.

(C) Volcano plot of differentially expressed lipid metabolites levels in human plasma after extraction of impacted mandibular third molar.

(D) Histogram showing the proportion of differentially expressed lipid metabolites in Fig1I-J.

(E) Bubble plots showing the proportion of differentially expressed lipid metabolites with least P value.

(F) Representative violin plots of differentially expressed lipid metabolites distribution in E.

(G) Matrix plots showing the correlation among metabolites in E.

(H) RaMP enrichment analysis of differentially expressed lipid metabolites levels in human plasma after extraction of impacted mandibular third molar.

Supplementary Fig. S3. Characterization of human dental follicle stem cells (hDFSCs).


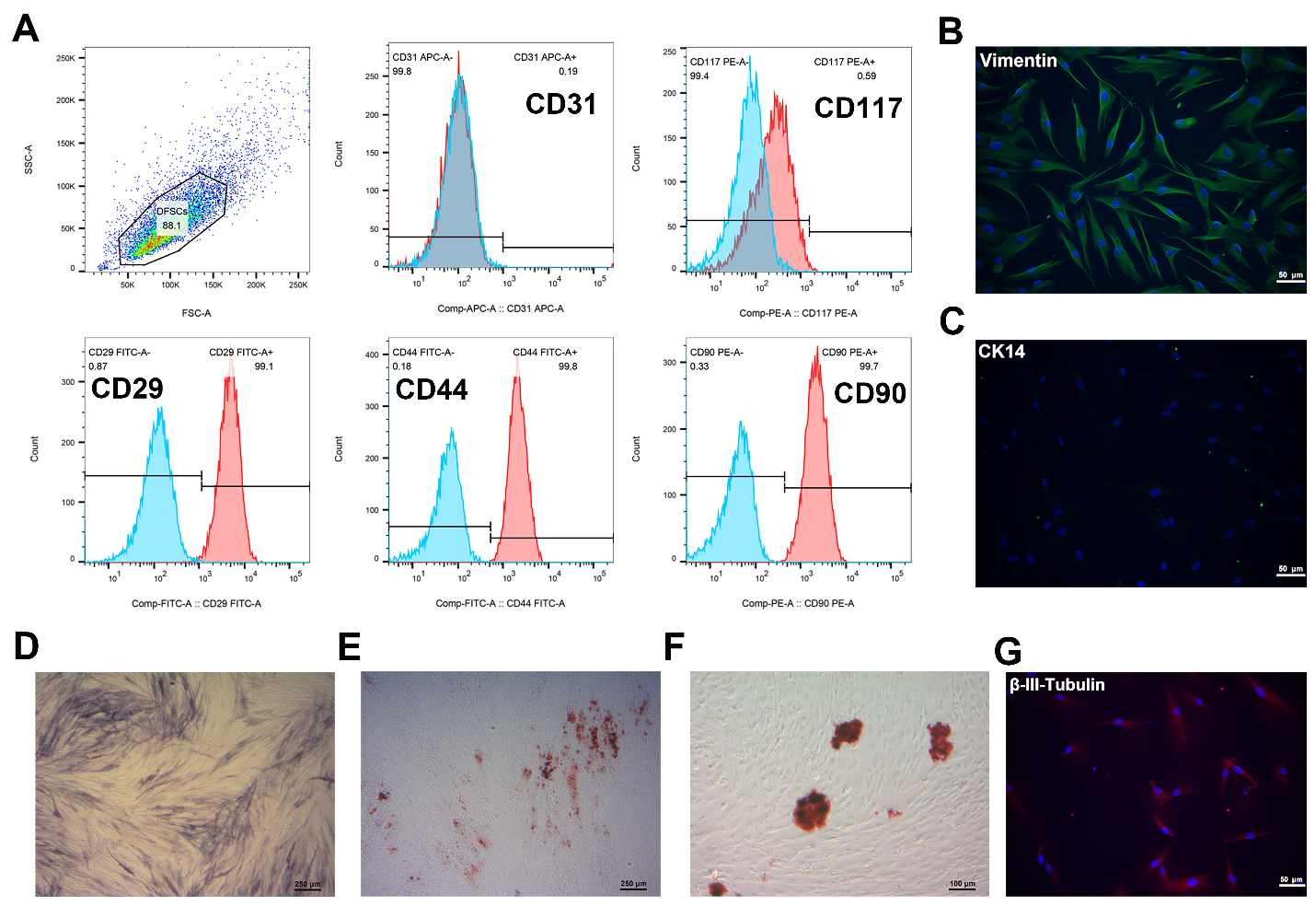


(A) Flow cytometry gating strategy. Positive markers including CD31, CD117. Negative markers involve CD29, CD44, CD90.

(B) Representative fluorescent imaging depicting that hDFSCs were stained for the mesenchymal marker (positive for Vimentin; green) and nuclei (DAPI; blue). Scale bar, 50 μm.

(C) Representative fluorescent imaging depicting that hDFSCs were stained for the epithelial marker (negative for CK14; green) and nuclei (DAPI; blue). Scale bar, 50 μm.

(D) Representative images of alkaline phosphatase staining for osteogenic differentiation after osteogenic culturing for 5 days. Scale bars: 250 μm.

(E) Representative images of alizarin red s staining for matrix mineralization after osteogenic culturing for 15 days. Scale bars, 200 μm.

(F) Representative images of oil red o staining for adipogenic differentiation after adipogenic induction for 15 days. Scale bars, 100 μm.

(G) Representative fluorescent imaging depicting that hDFSCs with neurogenic differentiation potential were stained for the neurogenic marker (positive for β-III-tubulin; red) and nuclei (DAPI; blue). Scale bar, 50 μm.

**Supplementary Fig. S4.**


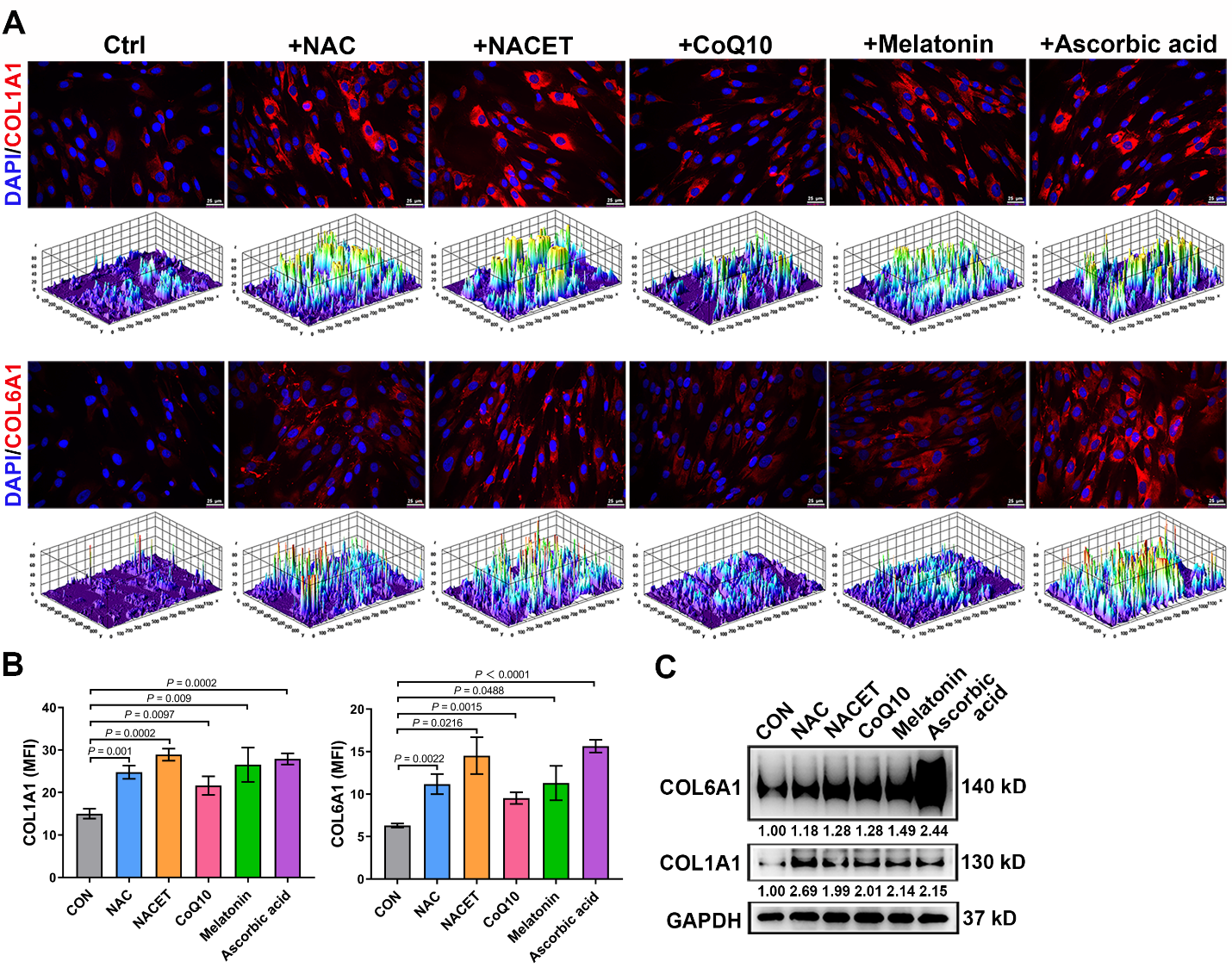


(A) Representative immunofluorescence staining of COL1A1 and COL6A1 in DFSCs after NAC, NACET, CoQ10, Melatonin or Ascorbic acid treatment. Scale bar, 25 μm.

(B) Quantification of immunofluorescence staining of COL1A1 and COL6A1 in A. *p <0.05 vs control; n = 3 independent experiments.

(C) Western blot analysis of COL1A1 and COL6A1 in DFSCs after NAC, NACET, CoQ10, Melatonin or Ascorbic acid treatment.

**Supplementary Fig. S5.**


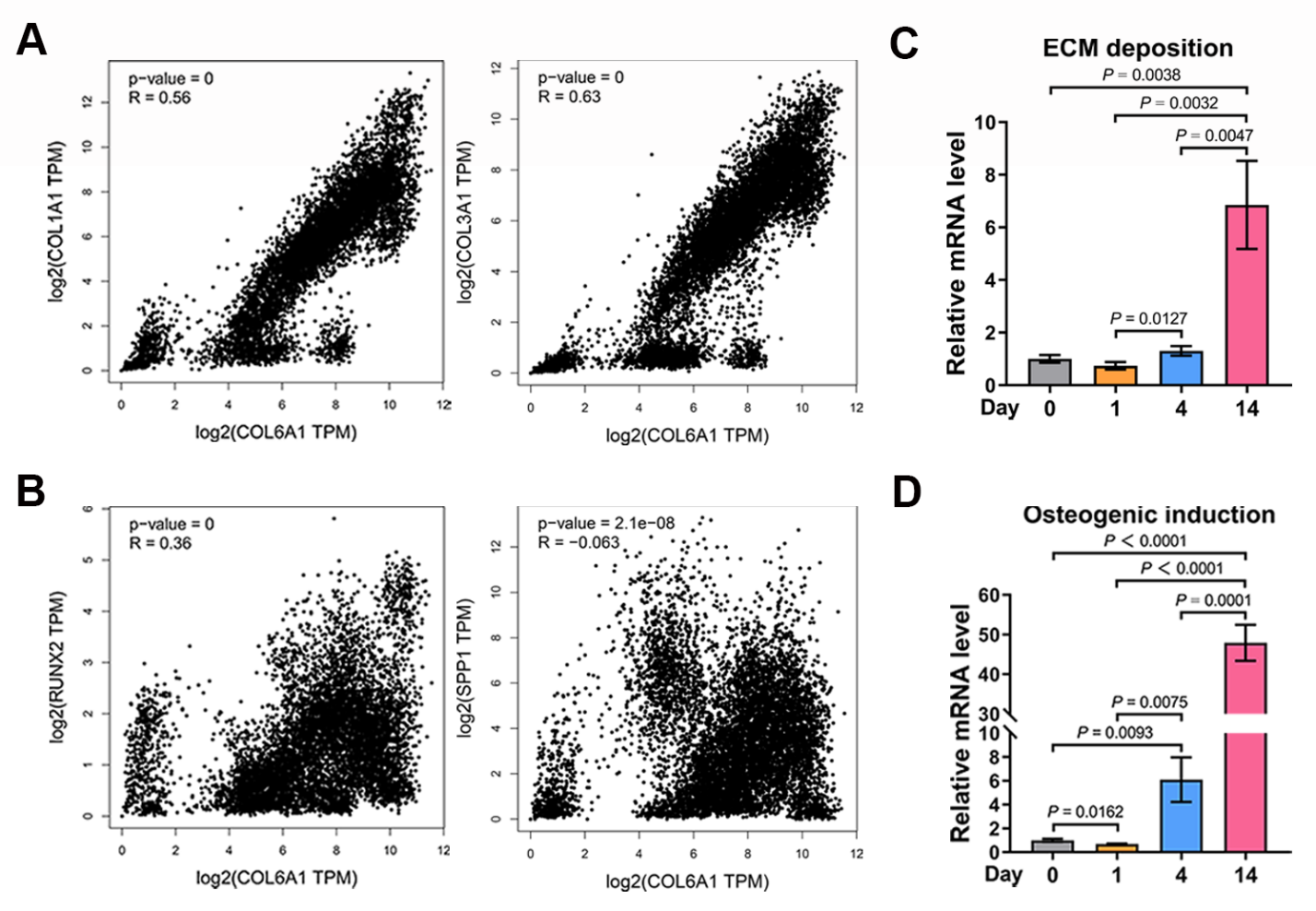


(A) Gene correlation analysis between COL6A1 and COL3A1. (http://gtexportal.org)

(B) Gene correlation analysis between COL6A1 and RUNX2, SPP1. (http://gtexportal.org)

(C) Relative mRNA levels of COL6A1 in DFSCs after ECM deposition for 0, 1, 4, 14 days. *p <0.05 vs control; n = 3 independent experiments.

(D) Relative mRNA levels of COL6A1 in DFSCs after osteogenic culturing for 0, 1, 4, 14 days. *p <0.05 vs control; n = 3 independent experiments.

**Supplementary Fig. S6.** **Characterization of rat dental follicle stem cells (rDFSCs).**


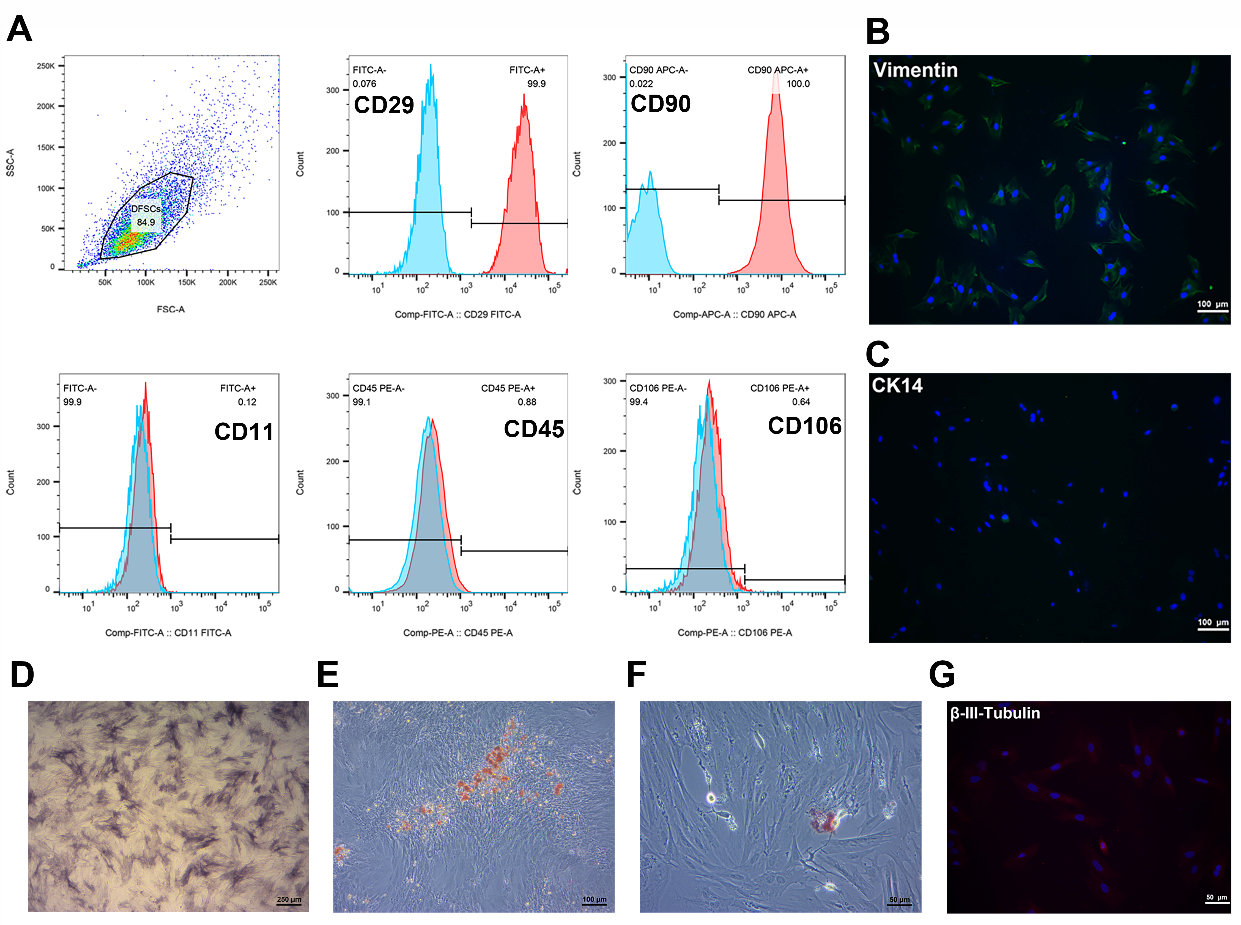


(A) Flow cytometry gating strategy. Positive markers including CD29, CD90. Negative markers involve CD11, CD45, CD106.

(B) Representative fluorescent imaging depicting that rDFSCs were stained for the mesenchymal marker (positive for Vimentin; green) and nuclei (DAPI; blue). Scale bar, 100 μm.

(C) Representative fluorescent imaging depicting that rDFSCs were stained for the epithelial marker (negative for CK14; green) and nuclei (DAPI; blue). Scale bar, 100 μm.

(D) Representative images of alkaline phosphatase staining for osteogenic differentiation after osteogenic culturing for 5 days. Scale bars, 250 μm.

(E) Representative images of alizarin red s staining for matrix mineralization after osteogenic culturing for 15 days. Scale bars, 100 μm.

(F) Representative images of oil red o staining for adipogenic differentiation after adipogenic induction for 15 days. Scale bars, 50 μm.

(G) Representative fluorescent imaging depicting that rDFSCs with neurogenic differentiation potential were stained for the neurogenic marker (positive for β-III-tubulin; red) and nuclei (DAPI; blue). Scale bar, 50 μm.

Supplementary Tab. S1 Antioxidant treatments.

| **Antioxidant** | **Catalogue number** | **Provider** | **Solvent** | **Stock concentration** | **Final concentration** | **Dilution ratio** |
| --- | --- | --- | --- | --- | --- | --- |
| N‐acetylcysteine (NAC) | A105422 | Aladdin, Shanghai, China | HEPES | 500 mM | 5 mM | 1:100 |
| N-Acetylcysteine ethyl ester (NACET) | R105876 | Rhawn, Shanghai, China | HEPES | 500 mM | 500 µM | 1:1000 |
| ubiquinone-10 (CoQ10) | C111044 | Aladdin, Shanghai, China | DMSO | 10 mM | 10 µM | 1:1000 |
| Melatonin | M118674 | Aladdin, Shanghai, China | DMSO | 100 mM | 100 µM | 1:1000 |
| Ascorbic acid | A8100 | Solarbio, Beijing, China | HEPES | 5 mg/ml | 50 µg/ml | 1:100 |

Supplementary Tab. S2 Primer sequences.

| **Primer** | **Sequence (5’-3’)** |
| --- | --- |
| GAPDH-homo-F | CTTTGGTATCGTGGAAGGACTC |
| GAPDH-homo-R | GTAGAGGCAGGGATGATGTTCT |
| COL1A1-homo-F | GAGGGCCAAGACGAAGACATC |
| COL1A1-homo-R | CAGATCACGTCATCGCACAAC |
| COL3A1-homo-F | TTGAAGGAGGATGTTCCCATCT |
| COL3A1-homo-R | ACAGACACATATTTGGCATGGTT |
| FN1-homo-F | CGGTGGCTGTCAGTCAAAG |
| FN1-homo-R | AAACCTCGGCTTCCTCCATAA |
| FBN1-homo-F | GCGGAAATCAGTGTATTGTCCC |
| FBN1-homo-R | CAGTGTTGTATGGATCTGGAGC |
| RUNX2-homo-F | TGGTTACTGTCATGGCGGGTA |
| RUNX2-homo-R | TCTCAGATCGTTGAACCTTGCTA |
| OPN-homo-F | GAAGTTTCGCAGACCTGACAT |
| OPN-homo-R | GTATGCACCATTCAACTCCTCG |
| COL6A1-homo-F | GACCTCGGACCTGTTGGGTAC |
| COL6A1-homo-R | TACCCCATCTCCCCCTTCAC |
| PIK3R1-homo-F | AAGAAGTTGAACGAGTGGTTGG |
| PIK3R1-homo-R | GCCCTGTTTACTGCTCTCCC |
| PIK3R2-homo-F | AAAGGCGGGAACAATAAGCTG |
| PIK3R2-homo-R | CAACGGAGCAGAAGGTGAGTG |
| PIK3R3-homo-F | CTGACATTTAATTCCGTGGTGGA |
| PIK3R3-homo-R | TCAAGTTTGGGATTGTACTGAGC |
| AKT1-homo-F | GTCATCGAACGCACCTTCCAT |
| AKT1-homo-R | AGCTTCAGGTACTCAAACTCGT |
| AKT2-homo-F | ACCACAGTCATCGAGAGGACC |
| AKT2-homo-R | GGAGCCACACTTGTAGTCCA |
| AKT3-homo-F | AATGGACAGAAGCTATCCAGGC |
| AKT3-homo-R | TGATGGGTTGTAGAGGCATCC |
| SOD1-homo-F | GGTGGGCCAAAGGATGAAGAG |
| SOD1-homo-R | CCACAAGCCAAACGACTTCC |
| SOD2-homo-F | TTTCAATAAGGAACGGGGACAC |
| SOD2-homo-R | GTGCTCCCACACATCAATCC |
| SOD3-homo-F | ATGCTGGCGCTACTGTGTTC |
| SOD3-homo-R | CTCCGCCGAGTCAGAGTTG |
| CAT-homo-F | TGGGATCTCGTTGGAAATAACAC |
| CAT-homo-R | TCAGGACGTAGGCTCCAGAAG |
| ITGA1-homo-F | CTGGACATAGTCATAGTGCTGGA |
| ITGA1-homo-R | ACCTGTGTCTGTTTAGGACCA |
| ITGA2-homo-F | CCTACAATGTTGGTCTCCCAGA |
| ITGA2-homo-R | AGTAACCAGTTGCCTTTTGGATT |
| ITGB1-homo-F | CCTACTTCTGCACGATGTGATG |
| ITGB1-homo-R | CCTTTGCTACGGTTGGTTACATT |

**Supplementary Tab. S3 GO enriched terms of biological process.**

| **Term ID** | **Term Desc and Full Name** | **Candidate Gene Number** | **Gene Number** | **Rich Ratio** | **P value** | **Q value** |
| --- | --- | --- | --- | --- | --- | --- |
| GO:0030198 | extracellular matrix organization | 40 | 246 | 0.162601626 | 3.00E-15 | 1.04E-11 |
| GO:0030199 | collagen fibril organization | 13 | 47 | 0.276595745 | 1.03E-08 | 1.79E-05 |
| GO:0051591 | response to cAMP | 12 | 51 | 0.235294118 | 2.70E-07 | 3.13E-04 |
| GO:0002042 | cell migration involved in sprouting angiogenesis | 8 | 24 | 0.333333333 | 1.54E-06 | 0.001068966 |
| GO:0003429 | growth plate cartilage chondrocyte morphogenesis | 7 | 17 | 0.411764706 | 1.35E-06 | 0.001068966 |
| GO:0014070 | response to organic cyclic compound | 18 | 132 | 0.136363636 | 1.97E-06 | 0.001137408 |
| GO:0071230 | cellular response to amino acid stimulus | 11 | 55 | 0.2 | 4.73E-06 | 0.002347896 |
| GO:0009612 | response to mechanical stimulus | 12 | 68 | 0.176470588 | 6.95E-06 | 0.002680743 |
| GO:0060337 | type I interferon signaling pathway | 12 | 68 | 0.176470588 | 6.95E-06 | 0.002680743 |
| GO:0001649 | osteoblast differentiation | 16 | 118 | 0.13559322 | 7.79E-06 | 0.002706299 |
| GO:0042493 | response to drug | 29 | 328 | 0.088414634 | 1.59E-05 | 0.005020594 |
| GO:0001957 | intramembranous ossification | 4 | 6 | 0.666666667 | 2.70E-05 | 0.007802733 |
| GO:0019216 | regulation of lipid metabolic process | 14 | 104 | 0.134615385 | 3.10E-05 | 0.008280685 |
| GO:0043065 | positive regulation of apoptotic process | 31 | 377 | 0.082228117 | 3.39E-05 | 0.008395928 |
| GO:0019221 | cytokine-mediated signaling pathway | 27 | 309 | 0.087378641 | 3.81E-05 | 0.008813772 |
| GO:0045599 | negative regulation of fat cell differentiation | 9 | 46 | 0.195652174 | 4.19E-05 | 0.009085896 |
| GO:0001934 | positive regulation of protein phosphorylation | 20 | 200 | 0.1 | 6.03E-05 | 0.01179047 |
| GO:0009314 | response to radiation | 8 | 38 | 0.210526316 | 6.45E-05 | 0.01179047 |
| GO:0010628 | positive regulation of gene expression | 36 | 482 | 0.074688797 | 6.21E-05 | 0.01179047 |
| GO:0060394 | negative regulation of pathway-restricted SMAD protein phosphorylation | 5 | 13 | 0.384615385 | 7.09E-05 | 0.0123075 |
| GO:0008285 | negative regulation of cell proliferation | 34 | 452 | 0.075221239 | 8.62E-05 | 0.01424384 |
| GO:0043433 | negative regulation of DNA-binding transcription factor activity | 11 | 75 | 0.146666667 | 9.87E-05 | 0.01438549 |
| GO:0051726 | regulation of cell cycle | 17 | 159 | 0.106918239 | 9.31E-05 | 0.01438549 |
| GO:0071260 | cellular response to mechanical stimulus | 12 | 88 | 0.136363636 | 9.94E-05 | 0.01438549 |
| GO:0007155 | cell adhesion | 45 | 672 | 0.066964286 | 1.08E-04 | 0.01439581 |
| GO:0099054 | presynapse assembly | 5 | 14 | 0.357142857 | 1.07E-04 | 0.01439581 |
| GO:0007179 | transforming growth factor beta receptor signaling pathway | 13 | 104 | 0.125 | 1.28E-04 | 0.01647734 |
| GO:0010506 | regulation of autophagy | 11 | 79 | 0.139240506 | 1.59E-04 | 0.01943865 |
| GO:1902895 | positive regulation of pri-miRNA transcription by RNA polymerase II | 8 | 43 | 0.186046512 | 1.62E-04 | 0.01943865 |
| GO:0043066 | negative regulation of apoptotic process | 39 | 567 | 0.068783069 | 1.79E-04 | 0.02044244 |
| GO:0043537 | negative regulation of blood vessel endothelial cell migration | 6 | 24 | 0.25 | 1.98E-04 | 0.02044244 |
| GO:0045071 | negative regulation of viral genome replication | 8 | 44 | 0.181818182 | 1.92E-04 | 0.02044244 |
| GO:0045944 | positive regulation of transcription by RNA polymerase II | 71 | 1240 | 0.057258065 | 1.86E-04 | 0.02044244 |
| GO:0060700 | regulation of ribonuclease activity | 3 | 4 | 0.75 | 2.00E-04 | 0.02044244 |
| GO:0031397 | negative regulation of protein ubiquitination | 9 | 56 | 0.160714286 | 2.07E-04 | 0.02050015 |
| GO:0016477 | cell migration | 22 | 256 | 0.0859375 | 2.46E-04 | 0.02374258 |
| GO:0001525 | angiogenesis | 23 | 275 | 0.083636364 | 2.66E-04 | 0.02434452 |
| GO:0030282 | bone mineralization | 8 | 46 | 0.173913043 | 2.65E-04 | 0.02434452 |
| GO:0001655 | urogenital system development | 5 | 17 | 0.294117647 | 3.01E-04 | 0.02528819 |
| GO:0007411 | axon guidance | 21 | 243 | 0.086419753 | 3.13E-04 | 0.02528819 |
| GO:0043588 | skin development | 8 | 47 | 0.170212766 | 3.09E-04 | 0.02528819 |
| GO:0060021 | roof of mouth development | 10 | 72 | 0.138888889 | 3.20E-04 | 0.02528819 |
| GO:0071404 | cellular response to low-density lipoprotein particle stimulus | 5 | 17 | 0.294117647 | 3.01E-04 | 0.02528819 |
| GO:1901653 | cellular response to peptide | 5 | 17 | 0.294117647 | 3.01E-04 | 0.02528819 |
| GO:0030334 | regulation of cell migration | 12 | 102 | 0.117647059 | 4.08E-04 | 0.03076793 |
| GO:0045926 | negative regulation of growth | 5 | 18 | 0.277777778 | 4.04E-04 | 0.03076793 |
| GO:0006048 | UDP-N-acetylglucosamine biosynthetic process | 4 | 11 | 0.363636364 | 5.11E-04 | 0.03355736 |
| GO:0007399 | nervous system development | 37 | 557 | 0.066427289 | 5.07E-04 | 0.03355736 |
| GO:0014009 | glial cell proliferation | 4 | 11 | 0.363636364 | 5.11E-04 | 0.03355736 |
| GO:0030335 | positive regulation of cell migration | 21 | 251 | 0.083665339 | 4.83E-04 | 0.03355736 |
| GO:0032964 | collagen biosynthetic process | 4 | 11 | 0.363636364 | 5.11E-04 | 0.03355736 |
| GO:0034097 | response to cytokine | 9 | 63 | 0.142857143 | 5.12E-04 | 0.03355736 |
| GO:0035914 | skeletal muscle cell differentiation | 8 | 50 | 0.16 | 4.78E-04 | 0.03355736 |
| GO:0008284 | positive regulation of cell proliferation | 37 | 558 | 0.066308244 | 5.25E-04 | 0.03372577 |
| GO:0071560 | cellular response to transforming growth factor beta stimulus | 9 | 64 | 0.140625 | 5.77E-04 | 0.03640727 |
| GO:0032870 | cellular response to hormone stimulus | 8 | 52 | 0.153846154 | 6.28E-04 | 0.03891767 |
| GO:0051091 | positive regulation of DNA-binding transcription factor activity | 13 | 123 | 0.105691057 | 6.75E-04 | 0.04114514 |
| GO:0033280 | response to vitamin D | 5 | 20 | 0.25 | 6.88E-04 | 0.041156 |
| GO:0033137 | negative regulation of peptidyl-serine phosphorylation | 6 | 30 | 0.2 | 7.22E-04 | 0.04247535 |
| GO:0032367 | intracellular cholesterol transport | 4 | 12 | 0.333333333 | 7.44E-04 | 0.04303974 |
| GO:0032496 | response to lipopolysaccharide | 17 | 190 | 0.089473684 | 7.69E-04 | 0.04379115 |
| GO:0050807 | regulation of synapse organization | 6 | 31 | 0.193548387 | 8.67E-04 | 0.0485607 |

Supplementary Tab. S4 KEGG enriched pathways.

| **Term** | **ID** | **Input number** | **Background number** | **P-Value** | **Corrected P-Value** |
| --- | --- | --- | --- | --- | --- |
| Protein digestion and absorption | hsa04974 | 18 | 90 | 3.90E-13 | 8.06E-11 |
| Human papillomavirus infection | hsa05165 | 28 | 330 | 3.22E-11 | 4.28E-09 |
| Pathways in cancer | hsa05200 | 34 | 530 | 3.01E-10 | 2.87E-08 |
| PI3K-Akt signaling pathway | hsa04151 | 27 | 354 | 6.51E-10 | 5.76E-08 |
| Signaling pathways regulating pluripotency of stem cells | hsa04550 | 17 | 140 | 1.88E-09 | 1.56E-07 |
| Axon guidance | hsa04360 | 18 | 181 | 1.13E-08 | 7.77E-07 |
| Breast cancer | hsa05224 | 16 | 147 | 2.31E-08 | 1.51E-06 |
| MAPK signaling pathway | hsa04010 | 22 | 295 | 3.60E-08 | 2.16E-06 |
| TGF-beta signaling pathway | hsa04350 | 13 | 94 | 3.79E-08 | 2.24E-06 |
| ECM-receptor interaction | hsa04512 | 12 | 86 | 1.16E-07 | 6.15E-06 |
| Focal adhesion | hsa04510 | 17 | 199 | 2.17E-07 | 1.12E-05 |
| mTOR signaling pathway | hsa04150 | 15 | 153 | 2.21E-07 | 1.12E-05 |
| Hepatocellular carcinoma | hsa05225 | 15 | 168 | 6.65E-07 | 2.92E-05 |
| Gastric cancer | hsa05226 | 14 | 149 | 8.90E-07 | 3.72E-05 |
| Metabolic pathways | hsa01100 | 52 | 1433 | 9.82E-07 | 4.01E-05 |
| Relaxin signaling pathway | hsa04926 | 13 | 130 | 1.15E-06 | 4.58E-05 |
| AGE-RAGE signaling pathway in diabetic complications | hsa04933 | 11 | 100 | 3.27E-06 | 0.000105797 |
| Proteoglycans in cancer | hsa05205 | 15 | 203 | 5.86E-06 | 0.000176933 |
| Hippo signaling pathway | hsa04390 | 13 | 154 | 6.45E-06 | 0.000191146 |
| Transcriptional misregulation in cancer | hsa05202 | 14 | 186 | 9.79E-06 | 0.000275572 |
| Insulin signaling pathway | hsa04910 | 12 | 137 | 1.03E-05 | 0.000285517 |
| Small cell lung cancer | hsa05222 | 10 | 93 | 1.09E-05 | 0.000301362 |
| Prostate cancer | hsa05215 | 10 | 97 | 1.53E-05 | 0.000404404 |
| Cushing syndrome | hsa04934 | 12 | 155 | 3.23E-05 | 0.000726496 |
| Colorectal cancer | hsa05210 | 9 | 86 | 3.66E-05 | 0.000795544 |
| TNF signaling pathway | hsa04668 | 10 | 112 | 4.79E-05 | 0.000980513 |
| Fluid shear stress and atherosclerosis | hsa05418 | 11 | 139 | 5.67E-05 | 0.001115106 |
| Melanoma | hsa05218 | 8 | 72 | 6.70E-05 | 0.001290723 |
| Mineral absorption | hsa04978 | 7 | 53 | 7.12E-05 | 0.001363722 |
| PPAR signaling pathway | hsa03320 | 8 | 76 | 9.50E-05 | 0.001721992 |
| EGFR tyrosine kinase inhibitor resistance | hsa01521 | 8 | 79 | 0.000121779 | 0.002075835 |
| Rap1 signaling pathway | hsa04015 | 13 | 210 | 0.000131898 | 0.002217788 |
| Osteoclast differentiation | hsa04380 | 10 | 128 | 0.000134929 | 0.002258539 |
| Parathyroid hormone synthesis, secretion and action | hsa04928 | 9 | 106 | 0.000161984 | 0.00264006 |
| Cellular senescence | hsa04218 | 11 | 160 | 0.000182448 | 0.002897338 |
| Insulin resistance | hsa04931 | 9 | 108 | 0.000184623 | 0.002919403 |
| Basal cell carcinoma | hsa05217 | 7 | 63 | 0.000190853 | 0.003005129 |
| Apoptosis | hsa04210 | 10 | 136 | 0.000214014 | 0.003232837 |
| Measles | hsa05162 | 10 | 138 | 0.000238947 | 0.003565974 |
| Rheumatoid arthritis | hsa05323 | 8 | 91 | 0.000298609 | 0.004300902 |
| Renal cell carcinoma | hsa05211 | 7 | 69 | 0.000318807 | 0.004539031 |
| Epstein-Barr virus infection | hsa05169 | 12 | 201 | 0.000323003 | 0.004581219 |
| AMPK signaling pathway | hsa04152 | 9 | 120 | 0.000382754 | 0.005116403 |
| Amoebiasis | hsa05146 | 8 | 95 | 0.000390854 | 0.005167732 |
| Choline metabolism in cancer | hsa05231 | 8 | 99 | 0.000505033 | 0.006318859 |
| Hepatitis C | hsa05160 | 10 | 155 | 0.000566698 | 0.006826503 |
| Toll-like receptor signaling pathway | hsa04620 | 8 | 104 | 0.000684251 | 0.007921113 |
| Endometrial cancer | hsa05213 | 6 | 58 | 0.000774599 | 0.008516006 |
| Jak-STAT signaling pathway | hsa04630 | 10 | 162 | 0.000781708 | 0.008543607 |
| HIF-1 signaling pathway | hsa04066 | 8 | 109 | 0.000911492 | 0.00957574 |
| Cytokine-cytokine receptor interaction | hsa04060 | 14 | 294 | 0.000919477 | 0.009624724 |
| Toxoplasmosis | hsa05145 | 8 | 113 | 0.001133747 | 0.011204799 |
| Acute myeloid leukemia | hsa05221 | 6 | 66 | 0.00143973 | 0.013209969 |
| Non-small cell lung cancer | hsa05223 | 6 | 66 | 0.00143973 | 0.013209969 |
| Type II diabetes mellitus | hsa04930 | 5 | 46 | 0.001738091 | 0.015090526 |
| Regulation of actin cytoskeleton | hsa04810 | 11 | 214 | 0.001784343 | 0.015420045 |
| Cell cycle | hsa04110 | 8 | 124 | 0.001973413 | 0.016368753 |
| Amino sugar and nucleotide sugar metabolism | hsa00520 | 5 | 48 | 0.002065028 | 0.01671818 |
| Kaposi sarcoma-associated herpesvirus infection | hsa05167 | 10 | 186 | 0.002080916 | 0.016810183 |
| Human T-cell leukemia virus 1 infection | hsa05166 | 11 | 219 | 0.00211879 | 0.017079007 |
| Endocrine resistance | hsa01522 | 7 | 98 | 0.002175998 | 0.017426744 |
| Cholesterol metabolism | hsa04979 | 5 | 50 | 0.002434674 | 0.018577513 |
| Wnt signaling pathway | hsa04310 | 9 | 160 | 0.002568197 | 0.019318668 |
| Glioma | hsa05214 | 6 | 75 | 0.002630557 | 0.019589481 |
| Chronic myeloid leukemia | hsa05220 | 6 | 76 | 0.002798137 | 0.020356661 |
| FoxO signaling pathway | hsa04068 | 8 | 132 | 0.002845034 | 0.020607013 |
| Hepatitis B | hsa05161 | 9 | 163 | 0.002889004 | 0.020685044 |
| MicroRNAs in cancer | hsa05206 | 13 | 299 | 0.002946303 | 0.021029528 |
| Arrhythmogenic right ventricular cardiomyopathy (ARVC) | hsa05412 | 6 | 77 | 0.002973579 | 0.021029528 |
| Complement and coagulation cascades | hsa04610 | 6 | 79 | 0.003348954 | 0.022834337 |
| Th17 cell differentiation | hsa04659 | 7 | 107 | 0.003453137 | 0.023458604 |
| Pathogenic Escherichia coli infection | hsa05130 | 5 | 55 | 0.003566066 | 0.024006343 |
| Regulation of lipolysis in adipocytes | hsa04923 | 5 | 55 | 0.003566066 | 0.024006343 |
| ErbB signaling pathway | hsa04012 | 6 | 85 | 0.004687411 | 0.029635944 |
| Longevity regulating pathway - multiple species | hsa04213 | 5 | 62 | 0.005723628 | 0.033319851 |
| Steroid biosynthesis | hsa00100 | 3 | 19 | 0.005951847 | 0.033961661 |
| Thyroid hormone signaling pathway | hsa04919 | 7 | 119 | 0.005958827 | 0.033961661 |
| Hypertrophic cardiomyopathy (HCM) | hsa05410 | 6 | 90 | 0.006073463 | 0.034462807 |
| Ferroptosis | hsa04216 | 4 | 40 | 0.006603777 | 0.037068933 |
| Glycosaminoglycan biosynthesis - chondroitin sulfate / dermatan sulfate | hsa00532 | 3 | 20 | 0.006758993 | 0.037375622 |
| Inflammatory bowel disease (IBD) | hsa05321 | 5 | 65 | 0.00688183 | 0.037998338 |
| IL-17 signaling pathway | hsa04657 | 6 | 93 | 0.007036087 | 0.038632474 |
| Lysosome | hsa04142 | 7 | 123 | 0.007038263 | 0.038632474 |
| Platelet activation | hsa04611 | 7 | 124 | 0.007329383 | 0.039050337 |
| Human cytomegalovirus infection | hsa05163 | 10 | 225 | 0.007423695 | 0.039050337 |
| Dilated cardiomyopathy (DCM) | hsa05414 | 6 | 96 | 0.008104486 | 0.040974515 |
| Central carbon metabolism in cancer | hsa05230 | 5 | 69 | 0.00866817 | 0.043236133 |
| Carbohydrate digestion and absorption | hsa04973 | 4 | 44 | 0.008975 | 0.044408919 |
| Prolactin signaling pathway | hsa04917 | 5 | 70 | 0.009160342 | 0.044907811 |
| Phosphatidylinositol signaling system | hsa04070 | 6 | 99 | 0.009284731 | 0.044907811 |
| NF-kappa B signaling pathway | hsa04064 | 6 | 100 | 0.009704036 | 0.045018973 |
| Influenza A | hsa05164 | 8 | 167 | 0.010599872 | 0.048823972 |

Supplementary Tab. S5 The top 10 associations between COL6A1 and human tissues based on experiments from TISSUES (<http://tissues.jensenlab.org/>).

| **Name** | **Source** | **Evidence** | **Confidence** |
| --- | --- | --- | --- |
| Lung | UniGene | 96 ESTs | ★★★★★ |
| Bone | UniGene | 69 ESTs | ★★★★☆ |
| Brain | UniGene | 60 ESTs | ★★★★☆ |
| Heart | UniGene | 57 ESTs | ★★★★☆ |
| Skin | UniGene | 52 ESTs | ★★★★☆ |
| Uterus | UniGene | 52 ESTs | ★★★★☆ |
| Placenta | UniGene | 51 ESTs | ★★★★☆ |
| Left atrium | Cardiac proteome | 2.3E10, 3.7E10, 1.8E10 | ★★★☆☆ |
| Intestine | UniGene | 44 ESTs | ★★★☆☆ |
| Ovary | HPM | 70 peptides | ★★★☆☆ |

Supplementary Tab. S6 The top 20 associations between COL6A1 and human diseases based on MalaCards (<http://www.malacards.org/>).

| **MCID** | **Name** | **MIFTS** | **Score** |
| --- | --- | --- | --- |
| BTH005 | Bethlem Myopathy 1 | 64 | 92.913 |
| ULL002 | Ullrich Congenital Muscular Dystrophy 1 | 60 | 52.262 |
| CLL043 | Collagen Vi-Related Dystrophies | 35 | 47.574 |
| MYP004 | Myopathy | 67 | 32.099 |
| MSC005 | Muscular Dystrophy | 66 | 17.312 |
| DWN001 | Down Syndrome | 71 | 15.699 |
| MSC165 | Muscular Dystrophy, Congenital, Lmna-Related | 66 | 14.263 |
| HRT032 | Heart Disease | 80 | 13.994 |
| LMB006 | Limb-Girdle Muscular Dystrophy | 56 | 13.671 |
| HYP068 | Hyperostosis | 47 | 11.208 |
| EHL001 | Ehlers-Danlos Syndrome | 64 | 10.473 |
| KRT007 | Keratoconus | 50 | 10.473 |
| TRC096 | Trichothiodystrophy | 55 | 10.473 |
| ATS331 | Autosomal Recessive Limb-Girdle Muscular Dystrophy | 42 | 10.342 |
| CRN025 | Corneal Dystrophy | 51 | 10.342 |
| AMY091 | Amyotrophic Lateral Sclerosis 1 | 87 | 10.29 |
| DST002 | Distal Arthrogryposis | 61 | 10.163 |
| OSS014 | Ossification of the Posterior Longitudinal Ligament of Spine | 46 | 9.736 |
| BCK002 | Beckwith-Wiedemann Syndrome | 61 | 8.83 |
| DFF006 | Diffuse Idiopathic Skeletal Hyperostosis | 45 | 8.231 |
